# Interleukin-15Rα-Sushi-Fc Fusion Protein Co-Hitchhikes Interleukin-15 and Pheophorbide A for Cancer Photoimmunotherapy

**DOI:** 10.3390/pharmaceutics17050615

**Published:** 2025-05-05

**Authors:** Zhe Li, Jiaojiao Xu, Hongzheng Lin, Sheng Yu, Jingwen Sun, Chen Zhang, Sihang Zhang, Tingting Li, Afeng Yang, Wei Lu

**Affiliations:** 1 School of Pharmacy & Minhang Hospital, Key Laboratory of Smart Drug Delivery Ministry of Education & State Key Laboratory of Molecular Engineering of Polymers, Fudan University, 826 Zhangheng Road, Shanghai 201203, China; 2 Quzhou Fudan Institute, 108 Minjiang Avenue, Quzhou 324002, China

**Keywords:** Fc fusion protein, interleukin-15, pheophorbide A, hitchhiking, photodynamic therapy

## Abstract

**Background**: Interleukin-15 (IL-15) stimulates the proliferation of natural killer cells or T cells, which, in combination with photodynamic therapy (PDT), has emerged as an effective strategy for cancer photoimmunotherapy. Instead of direct cytokine receptor activation, IL-15 necessitates first binding to the IL-15 receptor α chain subunit (IL-15Rα), followed by trans-presentation to the IL-15 receptor β/γ chain subunit on the effector cells for pharmacologic activation. Therefore, the delivery of IL-15 remains a major challenge owing to its short half-life, its lack of targeting activity, and the limited availability of IL-15Rα. **Methods**: A co-hitchhiking delivery approach using recombinant IL-15 (rIL-15) and a photosensitizer, pheophorbide A (PhA), is developed for enhanced combinatorial cancer immunotherapy with PDT. A recombinant IL-15Rα-sushi-Fc fusion protein (rILR-Fc) is designed to load rIL-15 through the IL-15Rα sushi domain, which mimics its trans-presentation. Moreover, the Fc moiety of rILR-Fc can load PhA based on its high binding affinity. **Results:** Through self-assembly, rILR-Fc/PhA/rIL-15 nanoparticles (NPs) are formulated to co-hitchhike PhA and rIL-15, which improves the tumor accumulation of PhA and rIL-15 through receptor-mediated transcytosis. Moreover, the nanoparticles prolong the blood half-life of rIL-15 but do not alter the elimination rate of PhA from the blood. The rILR-Fc/PhA/rIL-15 NPs effectively elicit potent systemic antitumor immunity and long-lasting immune memory against tumor rechallenge in model mice bearing orthotopic colon tumors. **Conclusions**: The enhanced antitumor therapeutic effect demonstrates that the co-hitchhiking delivery strategy, optimizing the pharmacokinetics of both the photosensitizer and IL-15, provides a promising strategy for combinatorial photodynamic and IL-15 immunotherapy.

## 1. Introduction

Photodynamic therapy (PDT) employs a photosensitizer, light, and oxygen to destroy cancer cells by generating cytotoxic reactive oxygen species, especially singlet oxygen [1]. PDT does not only use singlet oxygen to kill tumor cells or destroy tumor blood vessels but also elicits an antitumor immune response by initiating immunogenic cell death (ICD). ICD primes naive T cells by promoting dendritic cell (DC) maturation with the elevated presentation of tumor-specific antigens on DCs [2]. However, the PDT-induced antitumor response is usually insufficient, leading to tumor recurrence in clinics [3].

The therapeutic effects of PDT can be enhanced by interleukin-15 (IL-15), inducing stronger anticancer immunity [4,5]. IL-15, similarly to IL-2, stimulates the proliferation of T cells to induce cytotoxic T lymphocytes (CTLs) and memory phenotype CD8^+^ T cells. Moreover, IL-15 activates natural killer (NK) cells [6]. In contrast to IL-2, IL-15 does not consistently activate regulatory T cells (Tregs) or mediate activation-induced cell death (AICD) [7]. The mechanism by which IL-15 exerts its pharmacologic activity involves IL-15 specifically binding to the IL-15 receptor α chain subunit (IL-15Rα) expressed on the surfaces of DCs or monocytes. Then, the IL-15/IL-15Rα complex is trans-presented to neighboring NK or CD8^+^ T cells expressing only the IL-15 receptor β/γ chain subunit [8].

Despite the promising results of combination therapy with PDT and IL-15, IL-15 needs to be injected locally due to its short half-life, lack of targeting activity and the limited availability of IL-15Rα in vivo [4,5,8,9]. Therefore, the unsatisfactory bioavailability of IL-15 restricts its clinical application. Increasing the affinity to the IL-15 receptor β/γ chain subunit via an IL-15 mutant (IL-15N72D, residue substitution at position 72) has been proven to improve the biological activity of IL-15 in vitro [10]. The half-life and bioavailability of IL-15 in vivo can be increased by using IL-15 pre-associated with partial [11,12] (sushi domain [13], high-affinity IL-15-binding domain of IL-15Rα) or whole [14,15] IL-15Rα fused with the Fc domain to mimic IL-15 trans-presentation [16]. However, such a delivery system lacks tumor-targeting efficiency, resulting in local injection with large doses [17]. Although several strategies have been developed to increase the tumor-targeting activity of the cytokine, including the tumor-targeting fusion protein [18], the receptor-masking strategy [19], and the steric hindrance strategy [20], the introduction of functional moieties increases the complexity of the delivery system.

On the other hand, a key challenge for PDT is that photosensitizers are usually hydrophobic, with low tumor accumulation [21]. Nanoparticle-based drug delivery platforms have been proven to overcome these limitations [22]. However, most nanoparticle-based drug delivery systems demonstrate prolonged systemic circulation, resulting in the delayed clearance of encapsulated photosensitizers and elevated phototoxicity risks [23,24]. Our previous study has reported a delivery strategy termed the “IgG-hitchhiking” approach [25]. The nanocomplexes self-assemble via the high affinity of a porphyrin-structured photosensitizer, pheophorbide A (PhA), to immunoglobin G (IgG), increasing the tumor accumulation of PhA within the initial hours following intravenous (i.v.) injection without altering the blood elimination half-life [26]. Therefore, a significantly enhanced anticancer therapeutic effect can be achieved through PDT applied within the initial hours following the injection of the nanocomplex.

**Scheme 1:**
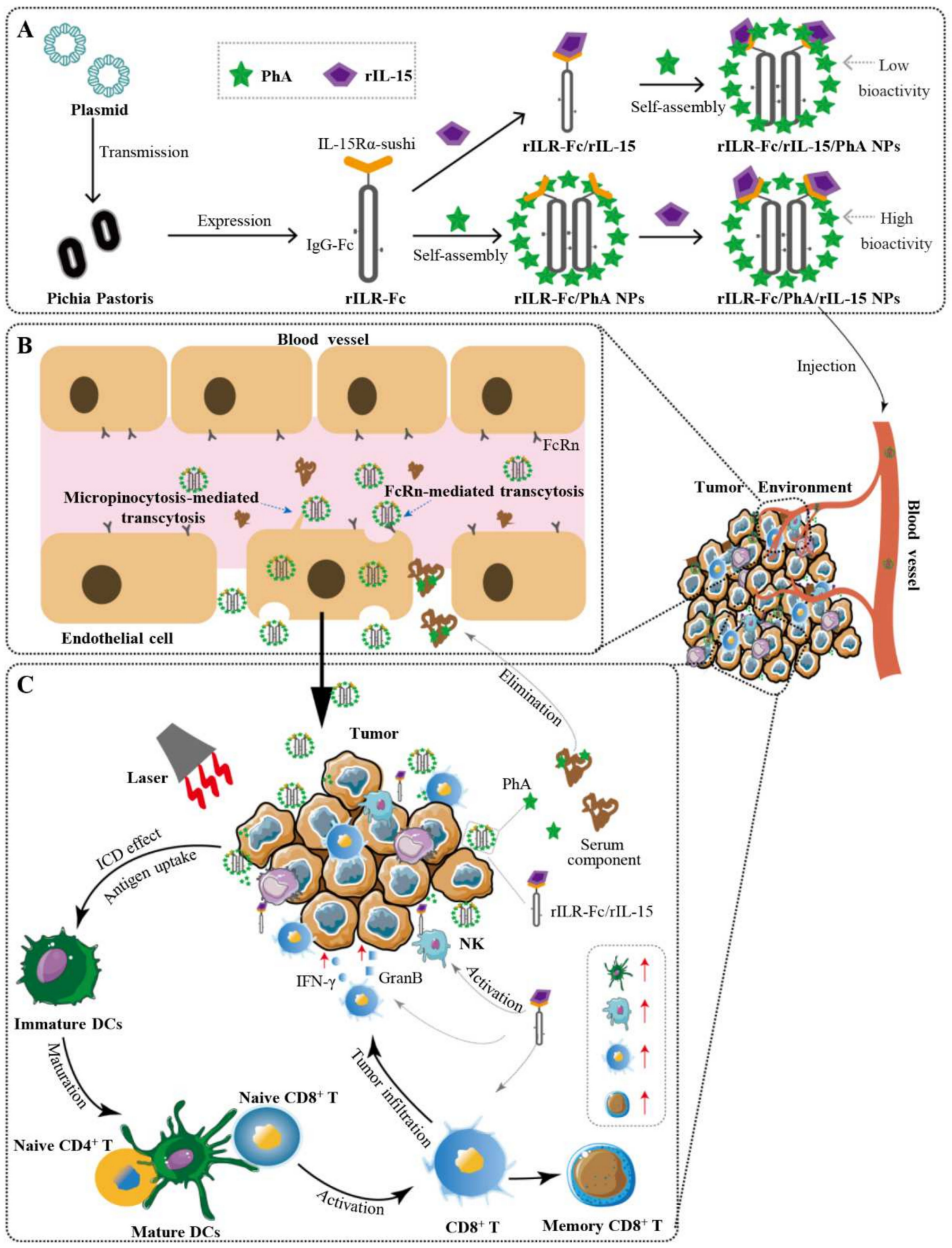
Schematic illustration of the Fc fusion protein co-hitchhiking interleukin-15 and pheophorbide A for enhanced cancer photoimmunotherapy. (**A**) The recombinant interleukin-15Rα-sushi-Fc fusion protein (rILR-Fc), in which the IL-15Rα-sushi domain is anchored onto the Fc domain of immunoglobin G (IgG-Fc), is genetically engineered. The rILR-Fc self-assembles into nanoparticles with pheophorbide A (PhA), followed by loading the recombinant interleukin-15 (rIL-15) to form rILR-Fc/PhA/rIL-15 NPs. (**B**) After systemic injection, the rILR-Fc/PhA/rIL-15 NPs accumulate in the tumor via neonatal Fc receptor (FcRn)-mediated transcytosis and micropinocytosis, followed by the quick elimination of PhA from the tumor. (**C**) rILR-Fc/PhA/rIL-15 NPs offer a combinatorial photodynamic and IL-15 immunotherapy, promoting the maturation of dendritic cells (DCs) and eliciting potent systemic antitumor immunity. ICD, immunogenic cell death. NK, natural killer. IFN-γ, interferon-gamma. GranB, Granzyme B.

In this work, we propose a co-hitchhiking strategy for delivering PhA and recombinant IL-15 (rIL-15) via an engineered recombinant protein consisting of the IL-15Rα-sushi domain fused with the Fc domain of IgG to enhance cancer photoimmunotherapy (Scheme 1). The recombinant interleukin-15Rα-sushi-Fc fusion protein (rILR-Fc) is used to prepare co-delivery nanocarriers, namely rILR-Fc/PhA/rIL-15 NPs, which can not only hitchhike PhA based on the high affinity of PhA to rILR-Fc but also carry rIL-15 through the receptor–ligand interaction to mimic its trans-presentation. The pharmacokinetic and distribution results show that the rILR-Fc/PhA/rIL-15 NPs facilitate the tumor accumulation of PhA within the first hour in mice bearing orthotopic colon cancer in comparison with free PhA, while the blood elimination half-life of PhA remains unchanged. Furthermore, the rILR-Fc/PhA/rIL-15 NPs prolong the blood half-life and enhance the tumor accumulation of rIL-15. Laser irradiation mediated by the rILR-Fc/PhA/rIL-15 NPs elicits potent systemic antitumor immunity. The co-hitchhiking nanoparticles provide a promising strategy for combinatorial photodynamic and IL-15 immunotherapy.

## 2. Materials and Methods

### 2.1. Materials

*E. coli* strain DH5α (Stratagene, Los Angeles, CA, USA) was used for the cloning experiments. For protein expression, *E. coli* strain BL21 (DE3) trxB (Yeasen, Shanghai, China) was used as the host strain, and the pET28a expression vector was obtained from Miaoling Biotechnology (Wuhan, China). The *Pichia pastoris* strain GS115 and pPIC9K expression vectors were purchased from Invitrogen (Carlsbad, CA, USA). KOD-plus-Neo, Nde I, Hind III, Not I, EcoR I, and Sac I were purchased from Toyobo (Osaka, Japan). The seamless assembly cloning kit was obtained from Clone Smarter (Houston, TX, USA).

### 2.2. Cells and Animals

The mouse CT26-Luc colon cancer cell line (CT26.WT-Fluc-Neo) was obtained from Imanis Life Sciences (Rochester, MN, USA). The M-07e cell line was provided by Procell Life Science & Technology Co., Ltd. (Wuhan, China). Human umbilical vein endothelial cells (HUVECs) were obtained from the American Type Culture Collection (Manassas, VA, USA). BALB/c mice (male, 6–8 weeks) and ICR mice (male, 6–8 weeks) were obtained from Shanghai Lingchang Biological Co., Ltd. (Shanghai, China). All animal experiments were performed in compliance with the guidelines established by the Institutional Animal Care and Use Committee (IACUC) of the School of Pharmacy, Fudan University.

### 2.3. Cloning, Expression, and Characterization of Recombinant Proteins

Previous studies demonstrated human IL-15-based immunotherapy boosted the antitumor cytotoxicity in multiple preclinical mouse tumor models [8,27,28]. The DNA sequence encoding N72D-mutated human IL-15 [10] or the human sushi domain (amino acids 1-77) of IL-15-Rα [13] fused with the CH2-CH3 domains of the human IgG1-Fc region was custom-synthesized by Sangon Biotech (Shanghai, China). rIL-15 was produced using the pET28a expression vector in *E. coli* BL21 (DE3) cells [29]. rILR-Fc was expressed by the *Pichia pastoris* expression system.

The protein of rIL-15 and rILR-Fc were analyzed by sodium dodecyl sulfate–polyacrylamide gel electrophoresis (SDS-PAGE) and a Western blot. The Western blot was performed according to our previously reported method [30]. The primary antibody used in the Western blot was the his-tag mouse monoclonal antibody (1:5000, 66005-1-Ig, Proteintech, Wuhan, China), and the secondary antibody was horseradish peroxidase (HRP)-conjugated goat anti-mouse IgG (1:5000, 33201ES60, Yeasen, Shanghai, China). The biological activity of rIL-15 and rILR-Fc/rIL-15 was determined by their ability to stimulate the proliferation of M-07e cells.

Further details of the production and confirmation of rIL-15 and rILR-Fc are provided in the Supplementary Materials.

### 2.4. Affinity Measurement

The dissociation constant (K_D_) between PhA and rILR-Fc was measured by the microscale thermophoresis (MST) technique, according to our previously reported method [26]. Briefly, PhA was dissolved in dimethyl sulfoxide (DMSO) and subjected to a series of dilutions to prepare ligand solutions ranging from 0.4 μM to 0.098 nM. Cy5-labeled rILR-Fc was prepared at a concentration of 15 nM and incubated with the ligand solutions for 10 min. The resulting samples were then loaded into capillaries (NanoTemper Technologies, Munich, Germany) for MST analysis.

### 2.5. Preparation and Characterization of Nanoparticles

Two methods were employed for the preparation of nanoparticles. PhA was dissolved in 0.1% NaOH (*w*/*v*) to achieve a concentration of 4 mg/mL. To prepare rILR-Fc/PhA/rIL-15 NPs, rILR-Fc (0.96 mg) was dissolved in 200 μL of phosphate-buffered saline (PBS). Then, 25 μL of PhA solution was added to the rILR-Fc solution under stirring at 500 rpm for 5 min. After stirring, the mixture (rILR-Fc/PhA NPs) was incubated with rIL-15 (12 μg) at 37 °C for 30 min. For the rILR-Fc/rIL-15/PhA NPs, rIL-15 (12 μg) was mixed with rILR-Fc (0.96 mg) and incubated at 37 °C for 30 min. Then, the mixture was dissolved in 200 μL of PBS. The prepared PhA solution (25 μL) was added to the above mixture under stirring at 500 rpm for 5 min. The size of the nanoparticles was measured by dynamic light scattering (DLS). The nanoparticles were observed via a transmission electron microscope (FEI Tecnai G2 20 TWIN, Hillsboro, OR, USA).

The biological activity of the nanoparticles was determined by their ability to stimulate the proliferation of M-07e cells, as described in the Supplementary Materials.

To test the encapsulation efficiency (EE) of the nanoparticles, they were ultrafiltrated (3 kDa) to remove free PhA. The concentration of free PhA was quantified by measuring the ultraviolet–visible (UV–Vis) absorption at 675 nm using a Hybrid Multi-Mode Microplate Reader (Biotek, Winooski, VT, USA). To isolate free rIL-15 from the nanoparticles, a Protein A agarose column was used to adsorb the nanoparticles. The concentration of free rIL-15 was quantified using an enzyme-linked immunosorbent assay (ELISA). Mouse anti-human IL-15 monoclonal antibody (MA5-23729) and rabbit anti-IL-15 polyclonal antibody (10360-T16) were obtained from Thermo Fisher (Carlsbad, CA, USA) and Sino Biological (Beijing, China), respectively. HRP-conjugated goat anti-rabbit IgG (34850ES60) was purchased from Yeasen (Shanghai, China). Then, 96-well plates were coated with mouse anti-human IL-15 monoclonal antibody (0.5 μg/mL) to capture rIL-15. After adding rIL-15 and incubating it at room temperature for 2 h, rabbit anti-IL-15 polyclonal antibody (1:7000) was added, followed by HRP-conjugated goat anti-rabbit IgG (1:7000). The EE was calculated using the following equation:

(1) $$\mathrm{EE}\;\left(\%\right)=\frac{\mathrm{Total}\;\mathrm{weight}\;\mathrm{of}\;\mathrm{PhA}\;\mathrm{or}\;\mathrm{rIL}\text{-}15\;-\;\mathrm{weight}\;\mathrm{of}\;\mathrm{free}\;\mathrm{PhA}\;\mathrm{or}\;\mathrm{rIL}\text{-}15}{\mathrm{Total}\;\mathrm{weight}\;\mathrm{of}\;\mathrm{PhA}\;\mathrm{or}\;\mathrm{rIL}\text{-}15}\;\times \;100\%$$

The in vitro release of PhA from the nanoparticles was performed in PBS or PBS containing 10% fetal bovine serum (FBS) at 37 °C. Nanoparticles containing 0.1 mg of PhA were incubated with 10% FBS for 0, 0.5, 1, 2, 4, and 6 h, respectively. The released PhA was separated by the Protein A agarose column that specifically absorbed rILR-Fc and nanoparticles. The eluent was collected, and the concentration of free PhA was determined by a Hybrid Multi-Mode Microplate Reader, as described above. For the in vitro stability assay, 100 μL of rILR-Fc/PhA/rIL-15 NPs was added to 10 mL of 10% FBS at 37 °C. The size distribution of the solution was measured by DLS at 0, 0.5, 2, 4, 8, or 12 h following mixture.

To analyze the cell-killing effect of PDT in vitro, CT26-Luc tumor cells were seeded in a 96-well cell culture plate and incubated at 37 °C in 5% CO_2_ for 24 h. Then, the CT26-Luc cells were incubated with rILR-Fc/PhA NPs or rILR-Fc/PhA/rIL-15 NPs for 2 h. After replacing the medium with fresh medium, the cells were irradiated with a 660 nm laser for 5 min at a power density of 20 mW/cm^2^. The cells were then cultured for another 24 h, followed by the 3-(4,5-dimethylthiazol-2-yl)-2,5-diphenyltetrazolium bromide (MTT) assay. Cell viability was calculated using the following equation:

(2) $$\mathrm{Cell}\;\mathrm{viability}\;(\%)=\frac{{A\;}_{\mathrm{test}}\;-\;{A\;}_{\mathrm{blank}}}{{A\;}_{\mathrm{control}}\;-\;A_{\;\mathrm{blank}}}\;\times \;100\%$$

### 2.6. Pharmacokinetic Analysis

Free PhA or rILR-Fc/PhA/rIL-15 NPs were administered via the tail vein into male BALB/c mice (6–8 weeks) at a dose of 2.5 mg/kg of PhA. The free PhA formulation was prepared by dissolving PhA in 0.1% NaOH (*w*/*v*) via sonication to obtain a 4 mg/mL stock solution, followed by dilution with PBS to obtain the injection dose. An alkali solution can increase the solubility of pheophorbide A applied to photodynamic therapy [31,32]. Blood samples were collected from the tail veins into heparin tubes at time points of 0.1, 0.5, 0.75, 1, 1.5, 2, 3, 4, and 5 h post-injection, respectively. PhA was extracted from plasma using a mixture of acetonitrile and methanol (1:1 *v*/*v*). The mixture was vortexed for 10 s, followed by centrifugation at 12,000 rpm for 10 min at 4 °C. The supernatant was transferred to a black 96-well plate, and the concentration of PhA was determined by the Hybrid Multi-Mode Microplate Reader (λ_ex_ = 400 nm, λ_em_ = 656 nm).

rIL-15, rILR-Fc/rIL-15 or rILR-Fc/PhA/rIL-15 NPs were injected via the tail vein into female C57BL/6 mice at a dose of 0.3 mg/kg of IL-15. For the rIL-15 group, blood was collected from the tail veins at time points of 5, 10, 15, 20, 30, 45, 60, 75, and 90 min post-injection, respectively. For the other groups, blood was collected at time points of 5, 10, 20, 30, 60, 90, 120, 180, 240, 300, and 360 min post-injection, respectively. The concentrations of rIL-15 in mouse sera were quantified by ELISA, as described above regarding the characterization of the nanoparticles.

Pharmacokinetic parameters including the elimination half-life (*t*_1/2_), the area under the concentration–time curve to the least measurable concentration (AUC_0→n_), the area under the moment curve (AUMC), the mean residence time (MRT), clearance (*Cl*), and the steady-state volume of distribution (*V*_ss_) were calculated using non-compartmental analysis.

### 2.7. Biodistribution Analysis

The tumor models were established according to our previous method [33]. For the subcutaneous (s.c.) tumor model, 2 × 10^6^ CT26-Luc cells in 50 μL PBS were implanted into the right axillary fossae of anesthetized male BALB/c mice. Orthotopic models were established through the injection of 2 × 10^6^ CT26-Luc cells (20 μL PBS) into the subserosal layer of the cecum.

When the s.c. tumor sizes reached 100 mm^3^, the CT26-Luc tumor-bearing mice were randomized into two groups receiving the i.v. administration of free PhA or rILR-Fc/PhA/rIL-15 NPs at equivalent PhA doses (2.5 mg/kg). Live fluorescence imaging was conducted using the IVIS system (Caliper Life Sciences, USA) (λ_ex_ = 675 nm, λ_em_ = 720 nm) at serial time points (18 min, 30 min, 45 min, 1 h, 1.5 h, 2 h, 3 h, 4 h, 6 h, 17 h, and 27 h) post-injection. The fluorescence intensity in the tumor region was quantified by deducting the fluorescence intensity of the mouse tumor before the administration of nanoparticles from that at each time point post-administration. For the ex vivo distribution of organs, the mice were euthanized at 0.5 h or 2 h after the injection of free PhA or rILR-Fc/PhA/rIL-15 NPs (2.5 mg/kg of PhA). The plasma, heart, liver, spleen, lung, kidney, lymph node, skin, and tumor were harvested for IVIS imaging (λ_ex_ = 675 nm, λ_em_ = 720 nm). The tissue uptake of PhA was presented through the semi-quantitative analysis of the fluorescence intensity [34].

To analyze the distribution of rIL-15, the CT26-Luc orthotopic model was established. Eight days after tumor cell inoculation, the mice were randomly divided into three groups and received an i.v. injection of rIL-15, rILR-Fc/rIL-15, or rILR-Fc/PhA/rIL-15 NPs at a dose of 1.5 mg/kg of rIL-15. The tissue was first flushed with PBS to remove residual blood and then weighed and cut into small pieces. Nine volumes of the radioimmunoprecipitation assay (RIPA) lysis buffer containing a protease inhibitor cocktail (Beyotime, Shanghai, China) were added to the tissue samples, followed by homogenization. The homogenate was centrifugated at 10,000 rpm for 10 min, and the supernatant was collected and stored at –80 °C for measurements. The rIL-15 concentrations in the serum and tissue of mice were determined by an ELISA, as described regarding the characterization of the nanoparticles, and the detection antibody was substituted with rabbit anti-IL-15 monoclonal antibody (MA5-43360, Thermo Fisher, Carlsbad, CA, USA). There were no exclusions of animals, experimental units, or data points reported.

### 2.8. Flow Cytometry Analysis of Cellular Uptake

The rILR-Fc was labeled with fluorescein isothiocyanate (FITC) prior to the rILR-Fc/PhA/rIL-15 NPs’ preparation. The cellular uptake and endocytosis inhibition experiments were performed on HUVECs. The cells were grown in a 24-well plate for 12 h and then incubated with FITC-labeled formulations. The cellular internalization of the formulation was determined by measuring the average intensity of FITC using flow cytometry at designated concentrations or predetermined incubation times. In the inhibition experiments on cellular uptake, the low-temperature inhibition experiment was performed at 4 °C in a refrigerator, and the endocytic inhibitors chlorpromazine (30 μM) or cytochalasin D (5 μM) were used to treat the HUVECs for 1 h prior to the addition of the FITC-labeled formulations. For the FcRn blocking experiment, the FITC-labeled formulations were incubated with staphylococcal protein A at an equivalent molar concentration for 30 min at 37 °C before incubation with HUVECs. The data were acquired and analyzed with a CytoFLEX flow cytometer (Beckman Coulter, Brea, CA, USA).

### 2.9. Intravital Fluorescence Microscopic Imaging

The extravasation of the rILR-Fc/PhA/rIL-15 NPs into the tumor was visualized by a home-built intravital fluorescence microscopic imaging system in the second near-infrared window (NIR-II), according to our previously reported method [26,33]. rILR-Fc was prelabeled with indocyanine green–N-hydroxysuccinimide ester (ICG-NHS) to prepare rILR-Fc/PhA/rIL-15 NPs. The tumor foci on the cecum were exposed and immobilized prior to imaging in the CT26-Luc orthotopic model. Then, the mice were i.v. injected immediately with rILR-Fc/PhA/rIL-15 NPs (0.9 mg/kg of rIL-15). The emission fluorescence of indocyanine green (ICG), excited by an 808 nm diode laser (Cnilaser, Changchun, China), was acquired with an InGaAs camera (TEKWIN, Xi’an, China). For the IgG blocking study, an excess amount of IgG (200 mg/kg, Solarbio, Beijing, China) was i.v. injected at 1 h prior to the administration of rILR-Fc/PhA/rIL-15 NPs. In the dual-channel intravital fluorescence microscopic imaging, a 660 nm diode laser (Cnilaser, Changchun, China) was added to excite PhA. The signals of PhA and ICG were collected with a scientific complementary metal oxide semiconductor (sCMOS) camera (PCO AG, Kelheim, Germany) and an InGaAs camera, respectively.

The degree of extravasation of nanoparticles from the blood vessels to the tumor parenchyma was compared using the area under the normalized fluorescence intensity–distance curve (AUNFIC), according to our previous studies [26,35]. Briefly, images from different fluorescent channels were processed by Photoshop CC 2023 (Adobe, San Jose, CA, USA) for pseudo-coloring. The fluorescence intensity of the acquired images was then normalized to the intensity of the blood vessel at 4 or 6 min after injection. The curve of the normalized fluorescence intensity over the diffusive distance was plotted, and the AUNFIC was calculated by integration.

### 2.10. Tumor Treatment and Assessment of Immune Response

In the orthotopic CT26-Luc tumor model, four days after the CT26-Luc tumor cells’ inoculation, the mice were randomly divided into five groups: the PBS group—mice treated with PBS; the rILR-Fc/rIL-15 group—mice injected with rILR-Fc/rIL-15 (0.3 mg/kg of rIL-15); the free PhA + PDT group—mice injected with free PhA (2.5 mg/kg) followed by laser treatment; the rILR-Fc/PhA NPs + PDT group—mice injected with rILR-Fc/PhA NPs (2.5 mg/kg of PhA) followed by laser treatment; the rILR-Fc/PhA/rIL-15 NPs + PDT group—mice injected with rILR-Fc/PhA/rIL-15 NPs (2.5 mg/kg of PhA + 0.3 mg/kg of rIL-15) followed by laser treatment. For the laser treatment, the abdominal cavity of the mouse was opened under anesthesia; then, the tumor foci on the cecum were exposed and irradiated with a 660 nm diode laser (80 mW/cm^2^) through a quartz optic fiber (0.9 mm diameter) for 10 min at 35 min post-injection. The abdominal cavity was sutured after the laser treatment. In the rILR-Fc/rIL-15 or rILR-Fc/PhA/rIL-15 NPs + PDT group, the mice received an additional i.v. injection of rILR-Fc/rIL-15 (0.3 mg/kg of rIL-15) on days 8, 11, and 14. The tumor burden was monitored via the bioluminescence signal using an IVIS system after the i.p. injection of D-luciferin (15 mg/mL, 200 μL).

For the rechallenge model, the CT26-Luc-rejected mice following rILR-Fc/PhA/rIL-15 NP treatment were inoculated with 5 × 10^5^ of CT26-luc in the right flank. Age-matched naive male BALB/c mice were used as the controls. The tumor volume and the bioluminescence signals were recorded to assess the antitumor effects following different treatments. The death point was defined as when the tumor volume reached 2000 mm^3^, calculated by the following equation:

(3) $$Vt=\frac{L\;\times \;W^{2}}{2}$$

where V_t_ is the tumor volume, L is the tumor length, and W is the tumor width.

Flow cytometry analysis was performed on samples including the tumor, spleen, or draining lymph nodes (DLNs) obtained from the orthotopic CT26-Luc tumor model. On day 10 following the above therapeutic assessment, the mice were euthanized. The single-cell samples were prepared from the tissue, followed by staining with different fluorescently conjugated antibodies. The protocols for both the cellular membrane staining and intracellular staining followed our previously reported method [36]. The tested items included CD8^+^ T cells, CTLs (CD8^+^IFN-γ^+^ T cells), CD8^+^GranB^+^ T cells, and matured DCs in DLN; CD4^+^ T cells, CD8^+^ T cells, CD8^+^GranB^+^ T cells, CTLs, myeloid-derived suppressor cells (MDSC), NK cells, CD69^+^ NK cells, GranB^+^ NK cells, and memory CD8^+^ T cells in the spleen; and CD8^+^ T cells, CD8^+^GranB^+^ T cells, CTLs, Tregs, and NK cells in the tumor. The antibodies used are listed in Table S1. The data were acquired and analyzed with a CytoFLEX flow cytometer.

### 2.11. Toxicity Assessment

rILR-Fc/PhA/rIL-15 NPs were i.v. injected into healthy ICR mice as described for the tumor treatment. The major organs (heart, liver, spleen, lung, and kidney) were collected and weighted at day 18. Whole blood and serum were collected for hematological and biochemical analysis. The organs were then fixed with 4% of paraformaldehyde to prepare paraffin-embedded slides for hematoxylin and eosin (H&E) staining. The organ coefficients were calculated using the following equation:

(4) $$\mathrm{Organ}\;\mathrm{coefficient}=\frac{\mathrm{organ}\;\mathrm{weight}}{\mathrm{body}\;\mathrm{weight}}\;\times \;100\%$$

To evaluate the skin photosensitivity, healthy BALB/c mice were depilated and randomly divided into three groups. The mice were i.v. injected with free PhA or rILR-Fc/PhA/rIL-15 NPs at a PhA dose of 2.5 mg/kg. Mice injected with PBS were used as controls. At 24 h post-injection, all mice were exposed to a long-arc xenon lamp (5 mW/cm^2^, 25 min) [26], which is generally used in solar simulation systems, emitting a high-intensity radiant flux with a visible spectrum closely approximating that of unfiltered daylight [37]. A 1.5 cm × 1.5 cm section of skin was collected from the dorsal areas of the mice for H&E staining.

### 2.12. Statistical Analysis

The statistical analysis was performed using the GraphPad Prism 8 software (GraphPad, San Diego, CA, USA). Statistical significance was determined by an unpaired two-tailed *t*-test, one-way ANOVA with Dunnett’s or Tukey’s multiple-comparisons post hoc test, and two-way ANOVA with Holm–Sidak’s multiple-comparisons post hoc test. Survival was analyzed using Kaplan–Meier survival curves. The curves were compared with the log-rank Mantel–Cox test. *p* < 0.05 was considered statistically significant.

## 3. Results and Discussion

### 3.1. Preparation and Characterization of rILR-Fc/PhA/rIL-15 NPs

**Figure 1:**
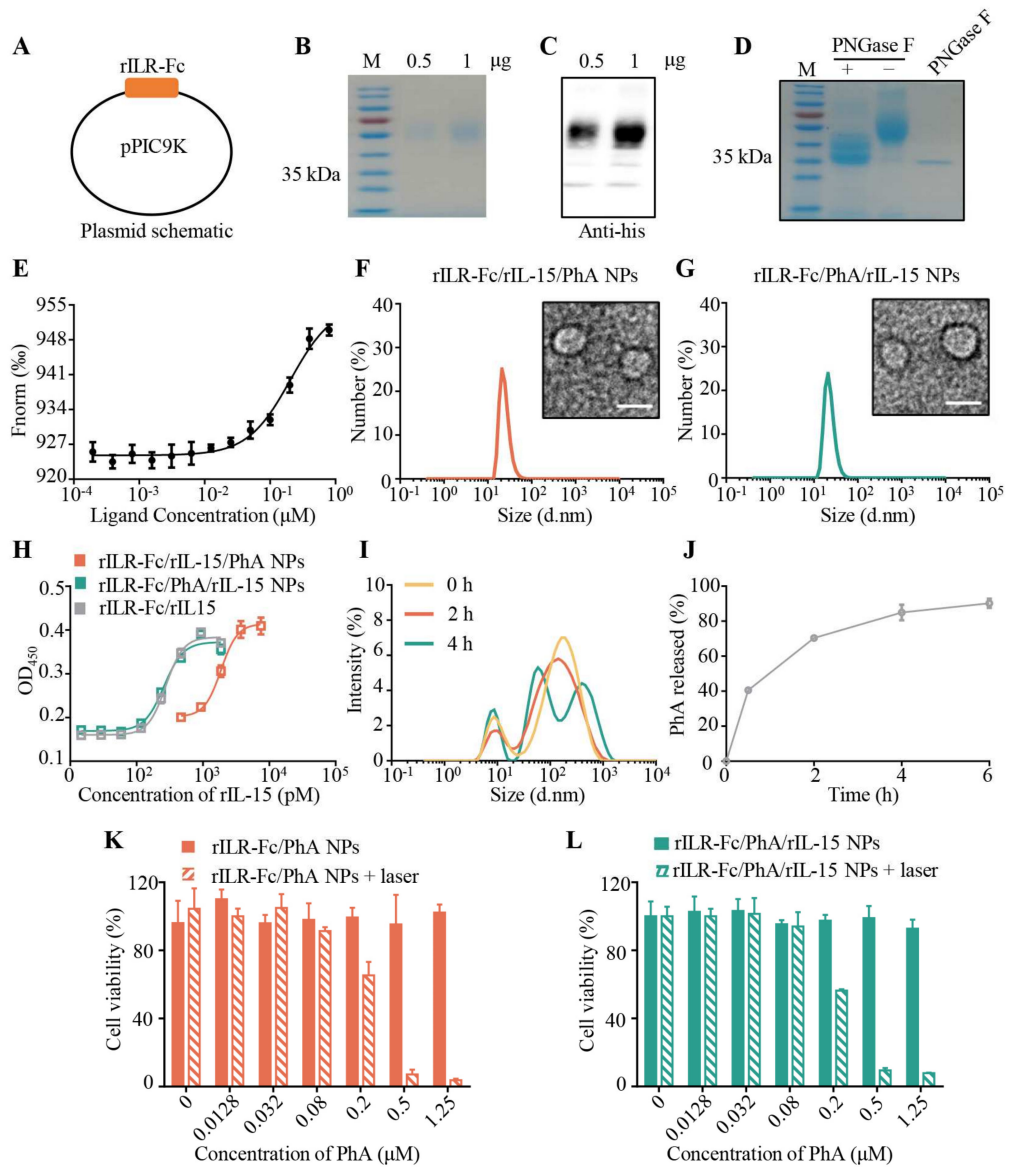
Preparation and characterization of rILR-Fc/PhA/rIL-15 NPs. (**A**) Schemes of the expression vector. (**B**,**C**) SDS-PAGE (**B**) and Western blot (**C**) analysis of rILR-Fc protein purified from the Pichia pastoris GS115 strain, respectively. (**D**) SDS-PAGE analysis of rILR-Fc protein deglycosylated with PNGase F. (**E**) Binding affinity of PhA to the Cy5-labeled rILR-Fc in PBS buffer, measured with microscale thermophoresis. The change in the normalized fluorescence (Fnorm, ‰) was analyzed. Data are means ± SD (*n* = 3). (**F**,**G**) Size distribution and transmission electron micrograph of rILR-Fc/rIL-15/PhA NPs (**F**) and rILR-Fc/PhA/rIL-15 NPs (**G**), respectively. Scale bar, 20 nm. (**H**) The bioactivity of rILR-Fc/PhA/rIL-15 NPs and rILR-Fc/rIL-15/PhA NPs detected by cell proliferation assays, respectively. rILR-Fc/rIL-15 protein was used as control. Data are means ± SD (*n* = 3). (**I**) Representative size distribution of rILR-Fc/PhA/rIL-15 NPs in 10% FBS for different time periods. (**J**) Cumulative release of PhA from rILR-Fc/PhA/rIL-15 NPs in 10% FBS over time. Data are means ± SD (*n* = 3). (**K**,**L**) Cell viability of CT26-Luc cells incubated with rILR-Fc/PhA NPs (**K**) or rILR-Fc/PhA/rIL-15 NPs (**L**), with or without laser irradiation (660 nm, 20 mW/cm^2^, 5 min), at 2 h post-incubation. After replacement with fresh medium, the cells were incubated for another 24 h for the MTT assay. Data are means ± SD (*n* = 3).

As shown in Figure 1A and Figure S1A, the designed plasmid pPIC9k-rILR-Fc expressed the Fc fusion protein containing the sushi domain of IL-15Rα in *Pichia pastoris*. After the expression vector pPIC9k-rILR-Fc was transfected to GS115, the recombinant fusion protein rILR-Fc was obtained in the culture medium. The rILR-Fc was confirmed by SDS-PAGE and Western blot experiments, respectively (Figure 1B,C). The rILR-Fc was glycosylated according to its molecular weight, ranging from 70 to 100 kDa. After deglycosylation with PNGase F, the molecular weight decreased to a range between 35 kDa and 50 kDa, which fit the calculated molecular weight of rILR-Fc (36.5 kDa, Figure 1D). Matrix-assisted laser desorption/ionization time-of-flight mass spectrometry (MALDI-TOF-MS) analysis showed that the molecular weight of rILR-Fc was 41,705 Da (Figure S1B), indicating that the glycosylated fusion protein was a monomer.

The bioactivity of rIL-15 generated in *E. coli* cells (Figure S2A–C) was tested for its effects on the proliferation of M-07e cells. As shown in Figure S2D, rIL-15 showed dose-dependent effects on the induction of M-07e cell proliferation. The half-effective concentration (EC_50_) of rIL-15 (0.92 nM) was ~3.5-fold smaller than that of the recombinant wild-type IL-15 (rIL-15-wt, 3.26 nM). Comparatively, the pre-association of rIL-15-wt with rILR-Fc or the commercial whole IL-15Rα fusion protein (rIL-15Rα-Fc) enhanced the biological activity; the EC_50_ was 1.65 nM for rIL-15Rα-Fc or 1.62 nM for rILR-Fc. These results demonstrated that the bioactivity of rILR-Fc was similar to that of rIL-15Rα-Fc when pre-associated with IL-15 to mimic its trans-presentation. Furthermore, as shown in Figure S3A,B, the affinity of rILR-Fc to rIL-15 was similar to that of rIL-15Rα-Fc to rIL-15 (0.240 nM versus 0.115 nM).

The K_D_ was calculated to be 0.2 μM based on MST (Figure 1E), demonstrating the high binding affinity of PhA to rILR-Fc. The two types of self-assembled nanoparticles—rILR-Fc/rIL-15/PhA NPs and rILR-Fc/PhA/rIL-15 NPs—with different loading sequences of rIL-15 and PhA exhibited similar particle sizes of 23.07 ± 0.58 nm and 24.48 ± 0.47 nm in diameter, respectively (Figure 1F,G). The transmission electron micrograph confirmed that the two types of nanoparticles had similar spherical nanostructures. To compare the bioactivity of these nanoparticles, the EC_50_ was measured for the proliferation of M-07e cells. The EC_50_ of rILR-Fc/PhA/rIL-15 NPs was similar to that of rILR-Fc/rIL-15 (0.67 nM versus 0.77 nM). However, the EC_50_ of rILR-Fc/rIL-15/PhA NPs (34.89 nM) was 52-fold higher than that rILR-Fc/PhA/rIL-15 NPs, indicating that the bioactivity of rIL-15 in the rILR-Fc/rIL-15/PhA NPs was significantly reduced during nanoparticles preparation (Figure 1H). As a result, rILR-Fc should be mixed with PhA to formulate rILR-Fc/PhA nanoparticles prior to rIL-15 loading. The rILR-Fc/PhA/rIL-15 NPs with high bioactivity of rIL-15 were used for further study.

The EE of PhA and rIL-15 for rILR-Fc/PhA/rIL-15 NPs was 98.94 ± 0.95% and 99.50 ± 0.05%, respectively (Table S2). The molar ratios of PhA and rIL-15 to rILR-Fc were further calculated to be 43.64 and 0.81, respectively. The results of the DLS analysis illustrated that the size distribution of rILR-Fc/PhA/rIL-15 NPs did not change in 10% FBS within 2 h, indicating that the rILR-Fc/PhA/rIL-15 NPs were stable in serum (Figure 1I and Figure S4A). The release profile of PhA showed that ~90% of PhA was released from the rILR-Fc/PhA/rIL-15 NPs in 10% FBS within 6 h (Figure 1J). The release rate of PhA in PBS without FBS was further measured at 0.5 h and 2 h (Figure S4B). Within the initial 0.5 h, the rILR-Fc/PhA/rIL-15 NPs released ~0.28% of PhA in PBS, while ~39% of PhA was released in 10% FBS. Similarly, the release of PhA in 10% FBS was much faster than in PBS within the initial 2 h. This result indicated that the serum components facilitated the dissociation of rILR-Fc/PhA/rIL-15 NPs.

Both rILR-Fc/PhA NPs and rILR-Fc/PhA/rIL-15 NPs did not significantly reduce the viability of the CT26-Luc cells at the incubation concentration of PhA up to 1.25 µM, indicating that the nanoparticles had good biocompatibility, without observable dark toxicity (Figure 1K,L). The half-maximal inhibitory concentrations (IC_50_) of rILR-Fc/PhA NPs and rILR-Fc/PhA/rIL-15 NPs following laser treatment were measured as 0.2240 µM and 0.2267 µM, respectively (Figure 1K,L). These data demonstrated that rILR-Fc/PhA NPs and rILR-Fc/PhA/rIL-15 NPs can significantly reduce the number of CT26-Luc tumor cells at a low concentration with an efficient PDT effect. At the same time, the rIL-15 bioactivity was not significantly changed following treatment with the tested laser power densities (Figure S4C).

### 3.2. Pharmacokinetics of rILR-Fc/PhA/rIL-15 NPs in Mice

**Figure 2:**
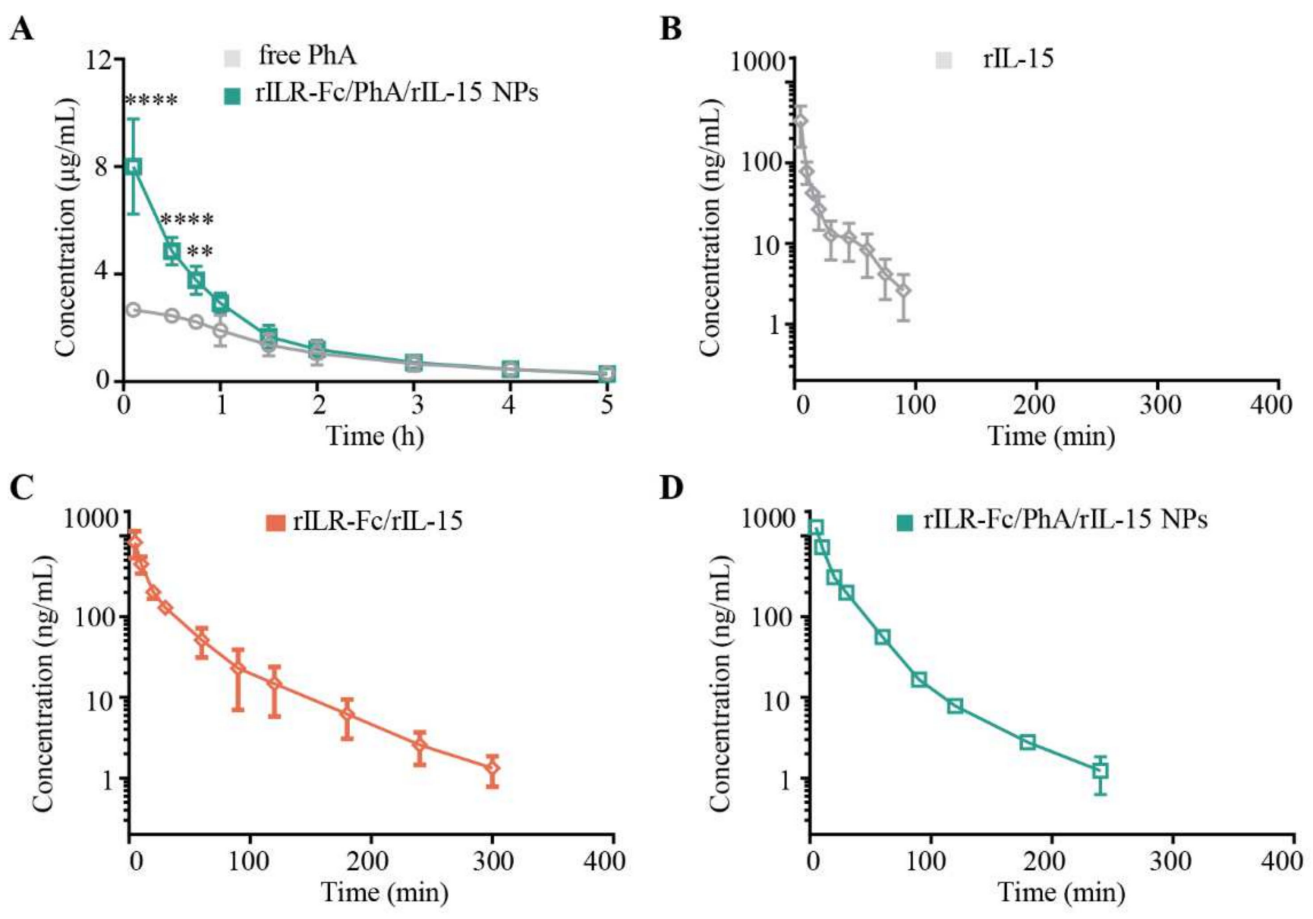
Pharmacokinetics of rILR-Fc/PhA/rIL-15 NPs. (**A**) Plasma PhA concentration–time curve following i.v. injection of free PhA or rILR-Fc/PhA/rIL-15 NPs in mice at a dose of 2.5 mg/kg of PhA. Data are means ± SD (*n* = 3). Statistical significance was calculated by two-way ANOVA with Holm–Sidak’s post hoc test. ** *p* < 0.01 and **** *p* < 0.0001 between the free PhA group and the rILR-Fc/PhA/rIL-15 NP group. (**B**–**D**) Pharmacokinetics of rIL-15 (**B**), rILR-Fc/rIL-15 (**C**), or rILR-Fc/PhA/rIL-15 NPs (**D**) following i.v. injection in mice at a dose of 0.3 mg/kg of rIL-15. Serum levels of rIL-15 were assessed by ELISA. Data are means ± SD (*n* = 3).

The plasma concentration–time curves of PhA in mice following the i.v. administration of free PhA or rILR-Fc/PhA/rIL-15 NPs were compared. The rILR-Fc/PhA/rIL-15 NPs exhibited PhA concentration levels that were significantly higher than those of free PhA within 45 min following injection (Figure 2A). However, the PhA concentration in the two groups did not show significant differences at or after 1 h post-injection. The plasma *t*_1/2_ and MRT did not have significant differences either (Table 1). Furthermore, the rILR-Fc/PhA/rIL-15 NPs significantly increased the AUC_0→n_ of PhA and decreased the *V*_ss_ of PhA compared with free PhA. These results demonstrated that the designed rILR-Fc/PhA/rIL-15 NPs, through the “IgG-hitchhiking” effect, exhibited the quick clearance of PhA from the blood circulation without changing its blood elimination half-life.

The rIL-15 concentration in the sera of mice was also determined following i.v. injection with rIL-15, rILR-Fc/rIL-15, or rILR-Fc/PhA/rIL-15 NPs (Figure 2B–D). The rILR-Fc/PhA/rIL-15 NPs and rILR-Fc/rIL-15 prolonged the blood circulation of rIL-15, resulting in a 1.95-fold or 2.88-fold increase in *t*_1/2_. The AUC_0→n_ was increased 4.11-fold or 3.35-fold. Compared to rILR-Fc/rIL-15, the serum *t*_1/2_ of rILR-Fc/PhA/rIL-15 NPs was decreased 0.68-fold, while the other pharmacokinetic parameters did not exhibit statistical significance (Table 2). These results indicated that rILR-Fc/PhA/rIL-15 NPs hitchhiking rIL-15 via the receptor–ligand interaction can prolong the blood half-life of rIL-15.

### 3.3. Enhanced Tumor Distribution of PhA and rIL-15 by rILR-Fc/PhA/rIL-15 NPs

**Figure 3:**
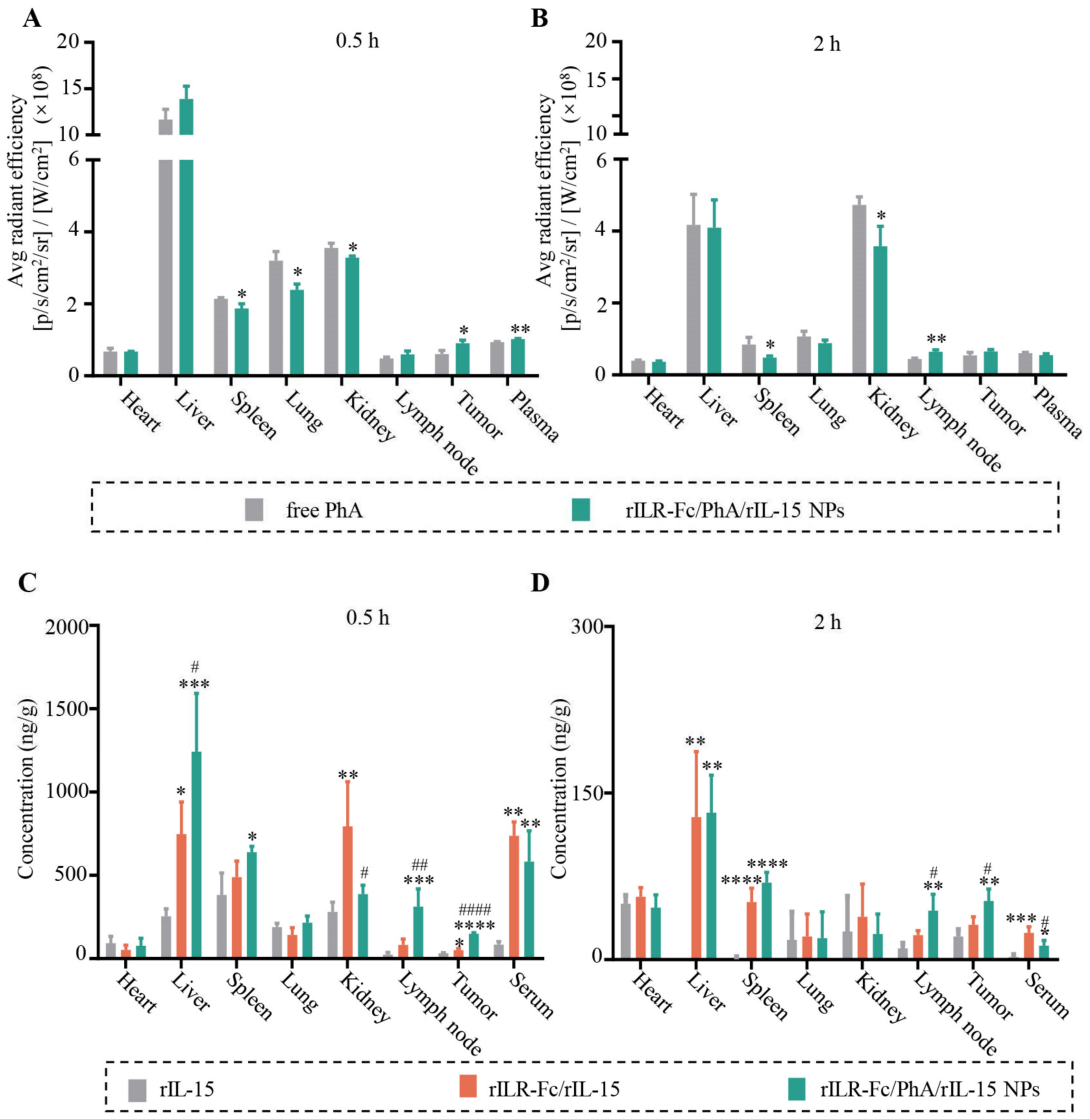
Biodistribution of PhA and rIL-15 from rILR-Fc/PhA/rIL-15 NPs in vivo. (**A**,**B**) Fluorescence intensity of PhA in different tissues by semi-quantification of the fluorescence imaging of mice bearing CT26-Luc orthotopic colon tumors at 0.5 h (**A**) or 2 h (**B**) after the i.v. injection of free PhA or rILR-Fc/PhA/rIL-15 NPs, as shown in Figure S6. Data are means ± SD (*n* = 3). Statistical significance was calculated by unpaired two-tailed *t*-test. * *p* < 0.05 and ** *p* < 0.01 compared with the free PhA group. (**C**,**D**) The concentrations of rIL-15 in various tissues in mice, measured by ELISA at 0.5 h (**C**) or 2 h (**D**) after i.v. injection. Data are means ± SD (*n* = 4). Statistical significance was calculated by one-way ANOVA with Tukey’s post hoc test. * *p* < 0.05, ** *p* < 0.01, *** *p* < 0.001, and **** *p* < 0.0001 compared with the rIL-15 group. ^#^ *p* < 0.05, ^##^ *p* < 0.01, and ^####^ *p* < 0.0001 compared with the rILR-Fc/rIL-15 group.

To test whether PhA could accumulate in tumor tissue, rILR-Fc/PhA/rIL-15 NPs or free PhA were injected into the tail veins of mice bearing s.c. CT26-Luc tumors. Live fluorescence imaging showed that the accumulation of PhA in the tumor reached a peak at 0.75 h and 1.5 h post-injection of rILR-Fc/PhA/rIL-15 NPs and free PhA, respectively (Figure S5A,B). Compared with free PhA, the rILR-Fc/PhA/rIL-15 NPs increased the fluorescence intensity of PhA 1.49-fold or 1.45-fold at 0.5 h or 0.75 h post-injection. At 1.5 h or later, the fluorescence intensity of the tumors did not have significant difference between the two groups. These results illustrate that the rILR-Fc/PhA/rIL-15 NPs facilitated the tumor accumulation of PhA during the first hour post-injection, followed by quick elimination from the tumor. Furthermore, we collected the organs to analyze the biodistribution of PhA in mice bearing orthotopic CT26-Luc colon tumors following the injection of free PhA or rILR-Fc/PhA/rIL-15 NPs (Figure S6). PhA was mainly distributed in the liver, spleen, lungs, and kidneys at 0.5 h post-injection, while the liver and kidneys were the major organs for PhA distribution at 2 h post-injection, respectively (Figure 3A,B). The fluorescence intensity of PhA in the tumors of the rILR-Fc/PhA/rIL-15 NPs group at 0.5 h was 1.48-fold that of the free PhA group. At 2 h post-injection, the rILR-Fc/PhA/rIL-15 NPs group did not show the significantly enhanced tumor accumulation of PhA in comparison with the free PhA group. These results were consistent with the live fluorescence imaging. The co-hitchhiking delivery approach operated similarly to our previously reported “IgG-hitchhiking” approach, which increased the tumor accumulation of the photosensitizer within the initial few hours after intravenous injection [26]. At later time points, such as 24 h post-injection, the “IgG-hitchhiking” approach did not show the enhanced accumulation of the photosensitizer in the tumor [25,26]. Therefore, the “hitchhiking” delivery strategy may not have the effect of enhanced permeability and retention.

We further determined the rIL-15 concentration in the major organs of mice i.v. injected with rIL-15, rILR-Fc/rIL-15, and rILR-Fc/PhA/rIL-15 NPs. As shown in Figure 3C,D, the rILR-Fc/PhA/rIL-15 NPs increased the rIL-15 accumulation in the tumor at 0.5 h post-injection by 4.1 and 2.6 times in comparison with rIL-15 and rILR-Fc/rIL-15, respectively. At 2 h post-injection, the concentrations of rIL-15 in the tumors of the rILR-Fc/PhA/rIL-15 NPs group were 2.6-fold and 1.7-fold higher than those of rIL-15 and rILR-Fc/rIL-15, respectively.

### 3.4. The FcRn-Mediated Transcytosis of rILR-Fc/PhA/rIL-15 NPs

**Figure 4:**
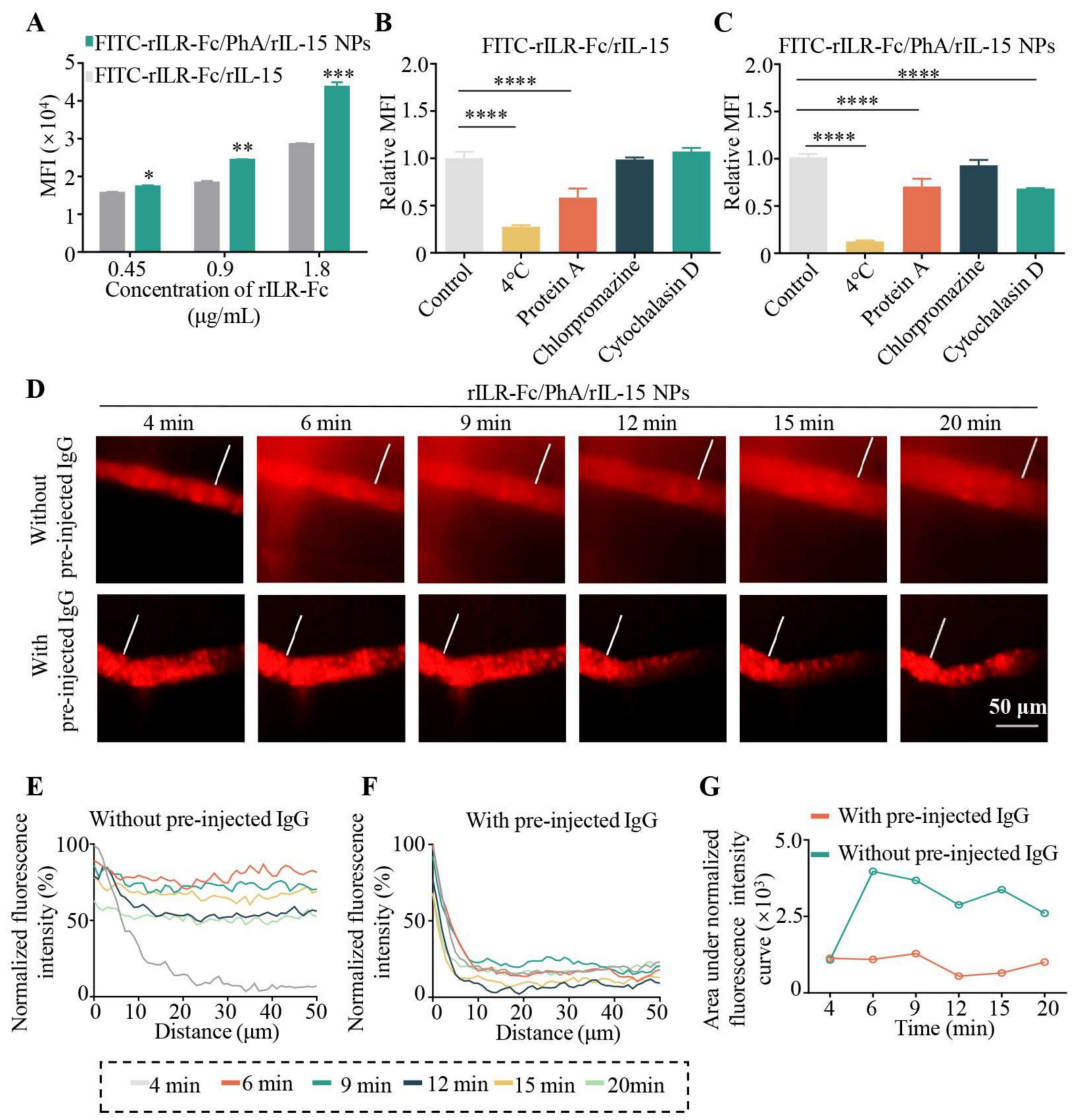
Extravasation of rILR-Fc/PhA/rIL-15 NPs across the tumor blood vessel. (**A**) Cellular uptake of the FITC-labeled rILR-Fc/rIL-15 or rILR-Fc/PhA/rIL-15 NPs by HUVECs after incubation for 1 h, as analyzed by flow cytometry. MFI, mean fluorescence intensity. Representative plots of flow cytometry are shown in Figure S7B. Data are means ± SD (*n* = 3). Statistical significance was calculated by two-way ANOVA with Holm–Sidak’s post hoc test. * *p* < 0.05, ** *p* < 0.01 and *** *p* < 0.001 compared with the FITC-rILR-Fc/rIL-15 group. (**B**,**C**) The uptake of rILR-Fc/rIL-15 (**B**) or rILR-Fc/PhA/rIL-15 NPs (**C**) by HUVECs, as analyzed by flow cytometry in the presence of various endocytosis inhibitors. The MFI of each group was normalized to that of the control group. Representative plots of flow cytometry are shown in Figure S7D. Data are means ± SD (*n* = 3). Statistical significance was calculated by one-way ANOVA with Tukey’s post hoc test. **** *p* < 0.0001. (**D**) Intravital fluorescence imaging of the extravasation of rILR-Fc/PhA/rIL-15 NPs at different times, with or without the pre-injection of an excessive amount of IgG in orthotopic CT26-Luc tumors. Red denotes the fluorescence signal of ICG-rILR-Fc. Bars, 50 μm. (**E**,**F**) Plots of the fluorescence intensity of ICG-rILR-Fc/PhA/rIL-15 NPs without (**E**) or with (**F**) blockade by IgG at various time points, as a function of the distance from the blood vessel, in a representative region marked by the white line in (**D**). (**G**) The AUNFICs of rILR-Fc/PhA/rIL-15 NPs at different times, with (**E**) or without (**F**) pre-injection with IgG.

We further investigated how the rILR-Fc/PhA/rIL-15 NPs enhanced the tumor accumulation of rIL-15. Transport across vascular endothelial cells of tumors via adsorptive-mediated transcytosis (AMT) or receptor-mediated transcytosis (RMT) is an essential process for the efficient delivery of anticancer agents [38,39]. The cellular uptake of rILR-Fc/PhA/rIL-15 NPs was evaluated by incubating HUVECs with the FITC-labeled nanoparticles or protein. The fluorescence intensity of FITC in the rILR-Fc/PhA/rIL-15 NPs was significantly higher than that of rILR-Fc/rIL-15 at various concentrations and different incubation times (Figure 4A and Figure S7A,B). Neither rILR-Fc/rIL-15 nor rILR-Fc/PhA/rIL-15 NPs exhibited cytotoxicity to HUVECs at the incubation concentrations of rILR-Fc (Figure S7C).

To understand the reasons for the high endocytosis rates of the nanoparticles, the cellular uptake was studied by treating HUVECs with specific endocytic inhibitors, including a low temperature of 4 °C for energy-dependent endocytosis [40], chlorpromazine for clathrin-mediated endocytosis [38], cytochalasin D for micropinocytosis-related endocytosis [41], and staphylococcal protein A for FcRn-mediated endocytosis [42]. Either low-temperature treatment or protein A blocking significantly inhibited the cellular uptake of the nanoparticles or protein compared with the control group (Figure 4B,C and Figure S7D). In contrast, cytochalasin D significantly inhibited the cellular uptake of the nanoparticles but not the protein. These results suggested that the cellular uptake of the rILR-Fc/PhA/rIL-15 NPs was an energy-dependent process, relying on FcRn-mediated endocytosis and micropinocytosis.

We utilized a home-built intravital NIR-II fluorescence microscopic imaging system to visualize the dynamics of the endothelial transcytosis process of the nanoparticles [33]. The fluorescence intensity of the ICG-labeled rILR-Fc/PhA/rIL-15 NPs in the tumor parenchyma was increased post-injection, reaching a peak at 6 min (Figure 4D). In comparison, with the FcRn receptors saturated by pre-injection with an excessive amount of free IgG [26], the signal of the ICG-labeled rILR-Fc/PhA/rIL-15 NPs was mainly distributed intravascularly (Figure 4D). To compare the degree of extravasation of nanoparticles from the blood vessels to the tumor parenchyma, a curve of the normalized fluorescence intensity over the diffusive distance was plotted, and the AUNFIC was calculated by integration [26,35]. The nanoparticles were transported across vascular endothelial cells of the tumor over time post-injection (Figure 4E–G). In contrast, the values of the AUNFIC were relatively low following pre-injection with free IgG. This result demonstrated that the transcytosis of rILR-Fc/PhA/rIL-15 NPs was inhibited through blocking the FcRn receptor. The enhanced tumor accumulation of rIL-15 could be attributed to the FcRn-mediated transcytosis of tumor vascular endothelial cells. Although the concentrations of rIL-15 in the tumors of the rILR-Fc/PhA/rIL-15 NP group were 2.6-fold those of the rIL-15 group at 2 h post-injection (Figure 3D), the specific types of immune cells that the nanoparticles can target remained to be investigated. Since the IL-15/IL-15Rα complex binds to the IL-15 receptor β/γ chain subunit through trans-presentation, the nanoparticles are believed to target NK or CD8^+^ T cells [8].

The disparity of extravasation in the tumor between PhA and rILR-Fc was also visualized with a dual-channel intravital fluorescence microscope (Figure S8A). The extravascular signal of PhA was increased within 45 min post-injection, followed by a decrease in both the intravascular and extravascular fluorescence intensities (Figure S8B–D). These data were consistent with the biodistribution results (Figure S5). The ICG-rILR-Fc signal was gradually decreased from the initial 6 min to 30 min post-injection (Figure S8B–D). After the extravasation of rILR-Fc, a rapid reduction in the rILR-Fc amount in the tumor appeared at about 6 min post-injection, while the tumor PhA level was continuously elevated within 45 min. This result indicated that PhA “hitchhiked on” the rILR-Fc molecule to facilitate extravasation from the tumor blood vessels to the tumor parenchyma in the early phase. Meanwhile, the quick elimination of PhA could be attributed to its “hitchhiking away” by the competitive binding of the serum components to PhA (Figure S4B).

### 3.5. Enhanced Antitumor Immune Response of PDT Plus rILR-Fc/PhA/rIL-15 NPs

**Figure 5:**
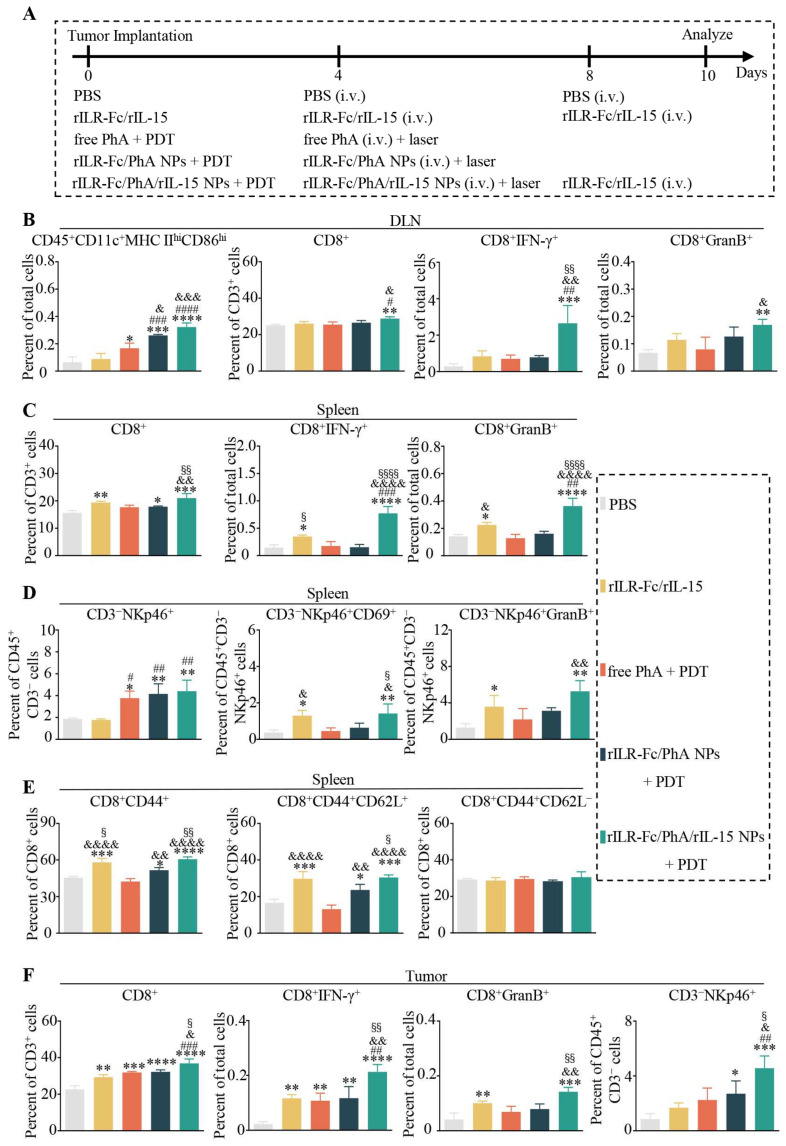
rILR-Fc/PhA/rIL-15 NPs elicited systemic anticancer immunity. (**A**) Scheme of therapeutic regimen for flow cytometry analysis of various immune cells. PBS group, mice treated with PBS. rILR-Fc/rIL-15 group, mice injected with rILR-Fc/rIL-15 (0.3 mg/kg of rIL-15). Free PhA + PDT group, mice injected with free PhA (2.5mg/kg) followed by laser treatment. rILR-Fc/PhA NPs + PDT group, mice injected with rILR-Fc/PhA NPs (2.5 mg/kg of PhA) followed by laser treatment. rILR-Fc/PhA/rIL-15 NPs + PDT group, mice injected with rILR-Fc/PhA NPs (2.5 mg/kg of PhA + 0.3 mg/kg of rIL-15) followed by laser treatment. For the laser treatment, the mice were irradiated with a 660 nm laser (80 W/cm^2^) for 10 min at 35 min post-injection. (**B**) Quantitative analysis of CD45^+^CD11c^+^MHCII^hi^CD86^hi^ cells, CD8^+^ T cells, CD8^+^IFN-γ^+^ T cells, and CD8^+^GranB^+^ T cells in DLN. Representative plots of flow cytometry are shown in Figure S10. (**C**–**E**) Quantitative analysis of CD8^+^ T cells, CD8^+^IFN-γ^+^ T cells, CD8^+^GranB^+^ T cells, CD45^+^CD3^−^NKp46^+^ cells, CD45^+^CD3^−-^NKp46^+^CD69^+^ cells, CD45^+^CD3^−^NKp46^+^GranB^+^ cells, CD8^+^CD44^+^ T cells (memory), CD8^+^CD44^+^CD62L^−^ T cells (effector memory), and CD8^+^CD44^+^CD62L^+^ T cells (central memory) in the spleen. Representative plots of flow cytometry are shown in Figure S11. (**F**) Quantitative analysis of CD8^+^ T cells, CD8^+^IFN-γ^+^ T cells, CD8^+^GranB^+^ T cells, and CD45^+^CD3^−^NKp46^+^ cells in tumors. Representative plots of flow cytometry are shown in Figure S12B. Data are means ± SD (*n* = 3). Statistical significance was calculated by one-way ANOVA with Dunnett’s post hoc test. * *p* < 0.05, ** *p* < 0.01, *** *p* < 0.001, and **** *p* < 0.0001 compared with the PBS group. ^#^ *p* < 0.05, ^##^ *p* < 0.01, ^###^ *p* < 0.001, and ^####^ *p* < 0.0001 compared with the rILR-Fc/rIL-15 group. ^&^ *p* < 0.05, ^&&^ *p* < 0.01, ^&&&^ *p* < 0.001, and ^&&&&^ *p* < 0.0001 compared with the free PhA + PDT group. ^§^ *p* < 0.05, ^§§^ *p* < 0.01, and ^§§§§^ *p* < 0.0001 compared with the rILR-Fc/PhA NPs + PDT group.

PDT induces ICD by triggering the rapid release of immunogenic signals such as calreticulin and high-mobility group box 1 (HMGB1), which activates immature DCs [2]. Here, the tumor DLNs were collected to examine the proportion of matured DCs (CD45^+^CD11c^+^MHCII^hi^CD86^hi^) by flow cytometry (Figure 5B). The rILR-Fc/PhA/rIL-15 NPs + PDT induced the highest amount of mature DCs among all groups, which was 4.98-fold higher than that of the PBS control. The combined effect on the upregulation of CD8^+^ T, CD8^+^GranB^+^ T cells, and CTLs in DLNs was observed following the treatment with rILR-Fc/PhA/rIL-15 NPs plus PDT (Figure 5B).

In the spleen, the rILR-Fc/PhA/rIL-15 NPs + PDT or rILR-Fc/rIL-15 significantly increased the percentage of CD8^+^ T cells, which was 1.34-fold higher than that of the PBS control (Figure 5C). The rILR-Fc/rIL-15 or rILR-Fc/PhA/rIL-15 NPs + PDT significantly increased the percentage of CD8^+^GranB^+^ T cells and CTLs compared with the PBS group (Figure 5C). The rILR-Fc/PhA/rIL-15 NPs + PDT increased the percentage of CTLs from 0.15% to 0.77% and that of CD8^+^GranB^+^ T cells from 0.14% to 0.36%. No significant effects on the expansion of CD4^+^ cells were observed (Figure S9A). All PDT treatment groups, including the free PhA + PDT, rILR-Fc/PhA NPs + PDT, and rILR-Fc/PhA/rIL-15 NPs + PDT groups, increased the percentage of NK (CD3^−^NKp46^+^) cells compared to the PBS control (Figure 5D). The activation marker (CD69^+^) and cytotoxic activation marker (GranB^+^) were only increased in the groups consisting of rILR-Fc/rIL-15 or rILR-Fc/PhA/rIL-15 NPs + PDT. These results demonstrated that the rILR-Fc/PhA/rIL-15 NPs plus PDT significantly induced antitumor immune responses. The rILR-Fc/PhA/rIL-15 NPs significantly increased the percentage of memory-phenotype T cells (CD8^+^CD44^+^), which was 1.32-fold higher than that of the PBS control (Figure 5E). Additionally, the rILR-Fc/PhA/rIL-15 NPs + PDT significantly increased the number of central memory cells (CD44^+^CD62L^+^) 1.83-fold compared with the control, but not the effector memory phenotype (CD44^+^CD62L^−^). The population of MDSCs was significantly reduced in the group of rILR-Fc/PhA/rIL-15 NPs + PDT compared to that in the control (Figure S9B). These results supported that rILR-Fc/PhA/rIL-15 NPs plus PDT significantly induced immune memory responses.

In tumors, rILR-Fc/PhA/rIL-15 NPs + PDT induced the largest amounts of CD8^+^ T cells, CTLs, or NK cells among all treated groups, respectively (Figure 5F). The rILR-Fc/PhA/rIL-15 NPs + PDT increased the percentages of CD8^+^ T cells, CTLs, and NK cells 1.63-fold, 9.22-fold, and 5.37-fold compared with the PBS control group, respectively. The percentage of CD8^+^GranB^+^ T cells was significantly increased, at 3.44-fold, in the group of rILR-Fc/PhA/rIL-15 NPs + PDT compared to that in the control. Furthermore, the percentage of T regulatory cells in the tumor was significantly decreased in all treatment groups compared with the PBS control (Figure S9C). These results demonstrated that the treatment with rILR-Fc/PhA/rIL-15 NPs plus PDT boosted the CTLs-mediated antitumor response for efficient immunotherapy.

### 3.6. Enhanced Antitumor Effects of PDT with rILR-Fc/PhA/rIL-15 NPs

**Figure 6:**
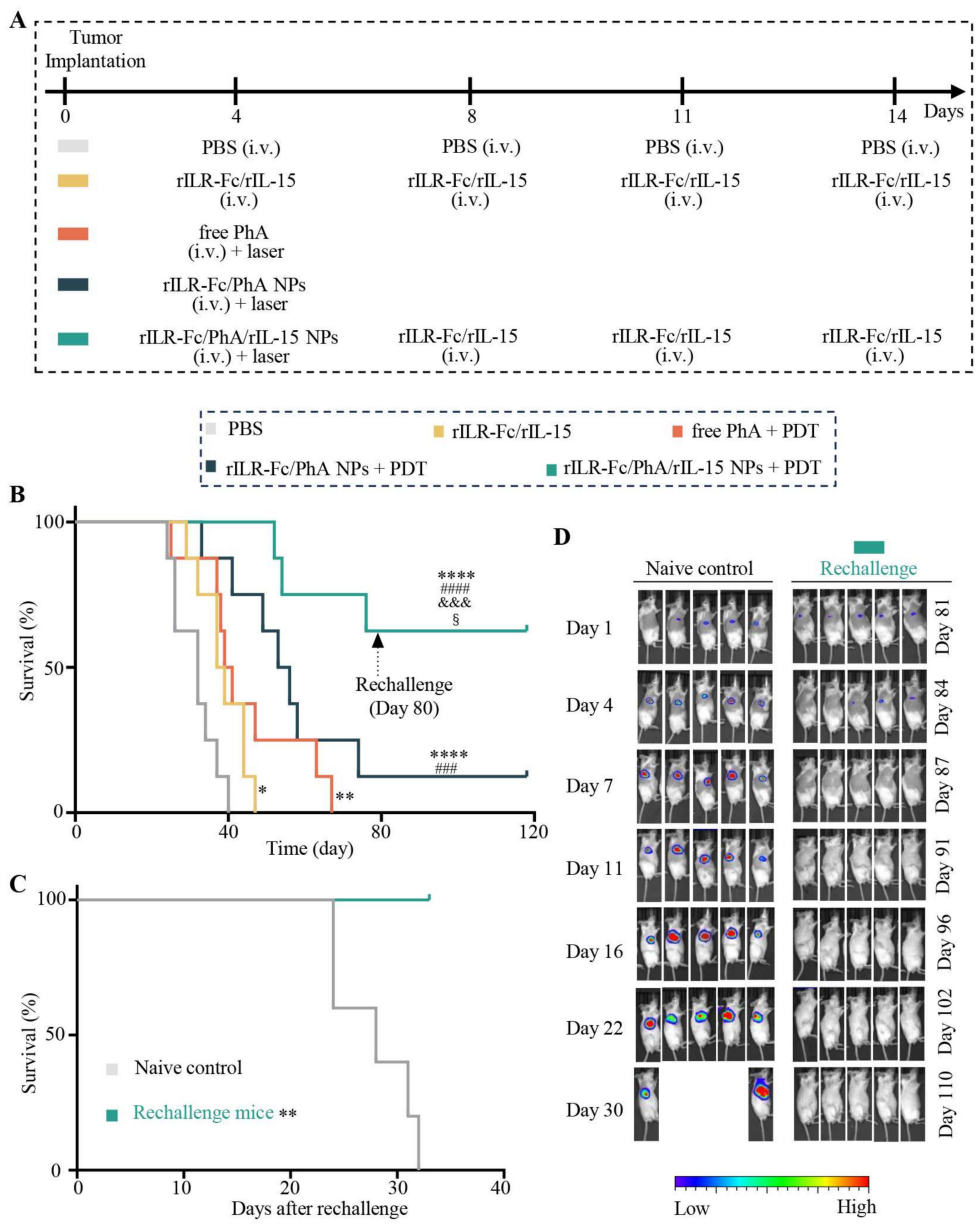
The therapeutic effect of rILR-Fc/PhA/rIL-15 NPs on mice bearing orthotopic CT26-Luc tumors. (**A**) Scheme of therapeutic regimen. PBS group, mice treated with PBS. rILR-Fc/rIL-15 group, mice injected with rILR-Fc/rIL-15 (0.3 mg/kg of rIL-15). Free PhA + PDT group, mice injected with free PhA (2.5 mg/kg) followed by laser treatment. rILR-Fc/PhA NPs + PDT group, mice injected with rILR-Fc/PhA NPs (2.5 mg/kg of PhA) followed by laser treatment. rILR-Fc/PhA/rIL-15 NPs + PDT group, mice injected with rILR-Fc/PhA NPs (2.5 mg/kg of PhA + 0.3 mg/kg of rIL-15) followed by laser treatment. For the laser treatment, the mice were irradiated with a 660 nm laser (80 W/cm^2^) for 10 min at 35 min post-injection. (**B**) Kaplan–Meier survival plots of the CT26-Luc-bearing mice after different treatments, as illustrated in (**A**). Significance was determined by log-rank analysis (*n* = 8). * *p* < 0.05, ** *p* < 0.01, and **** *p* < 0.001 compared with the PBS group. ^###^ *p* < 0.01, ^####^ *p* < 0.001 compared with the rILR-Fc/rIL-15 group. ^&&&^ *p* < 0.001 compared with the free PhA + PDT group. ^§^ *p* < 0.05 compared with the rILR-Fc/PhA NPs + PDT group. (**C**,**D**) Kaplan–Meier survival curve (**C**) and bioluminescence imaging (**D**) of rechallenged long-term survivors from (**B**) rILR-Fc/PhA/rIL-15 NPs + PDT group (*n* = 5). The tumor-free mice were inoculated with CT26-Luc cells (5 × 10^5^) subcutaneously at day 80. Naive control and naive BALB/c mice were inoculated with an equivalent quantity of CT26-Luc cells on the same day (*n* = 5). The tumor growth curves of these mice are shown in Figure S14. Significance was determined by log-rank analysis; ** *p* < 0.01 compared with the naive control group.

The analysis of the antitumor effect was carried out using mice bearing orthotopic CT26-Luc tumors, following various treatments (Figure 6A). Bioluminescence imaging was used to monitor tumor growth following the treatment. The tumor-bearing mice demonstrated a median survival time of 32 days in the PBS group (Figure 6B and Figure S13). The rILR-Fc/rIL-15 group (38 days) and the free PhA + PDT group (42 days) showed a slightly inhibitory effect on the tumor compared with the PBS control, respectively. However, the median survival time of the rILR-Fc/PhA NPs + PDT group was 54.5 days, significantly longer than that of the PBS or rILR-Fc/rIL-15 group, respectively (Figure 6B and Figure S13). Notably, five out of eight mice survived at day 80 without significant bioluminescence signals following treatment with the rILR-Fc/PhA/rIL-15 NPs plus PDT. Moreover, the five cured mice were completely protected against a subcutaneous CT26-Luc tumor challenge, without recurrence after the rechallenge for 32 days (Figure 6C,D and Figure S14). Taken together, these findings demonstrated that rILR-Fc/PhA/rIL-15 NPs plus PDT can induce a combinatorial antitumor effect, immunological memory, and long-term antitumor immunity, leading to resistance to tumor rechallenge.

### 3.7. Biocompatible rILR-Fc/PhA/rIL-15 NPs

**Figure 7:**
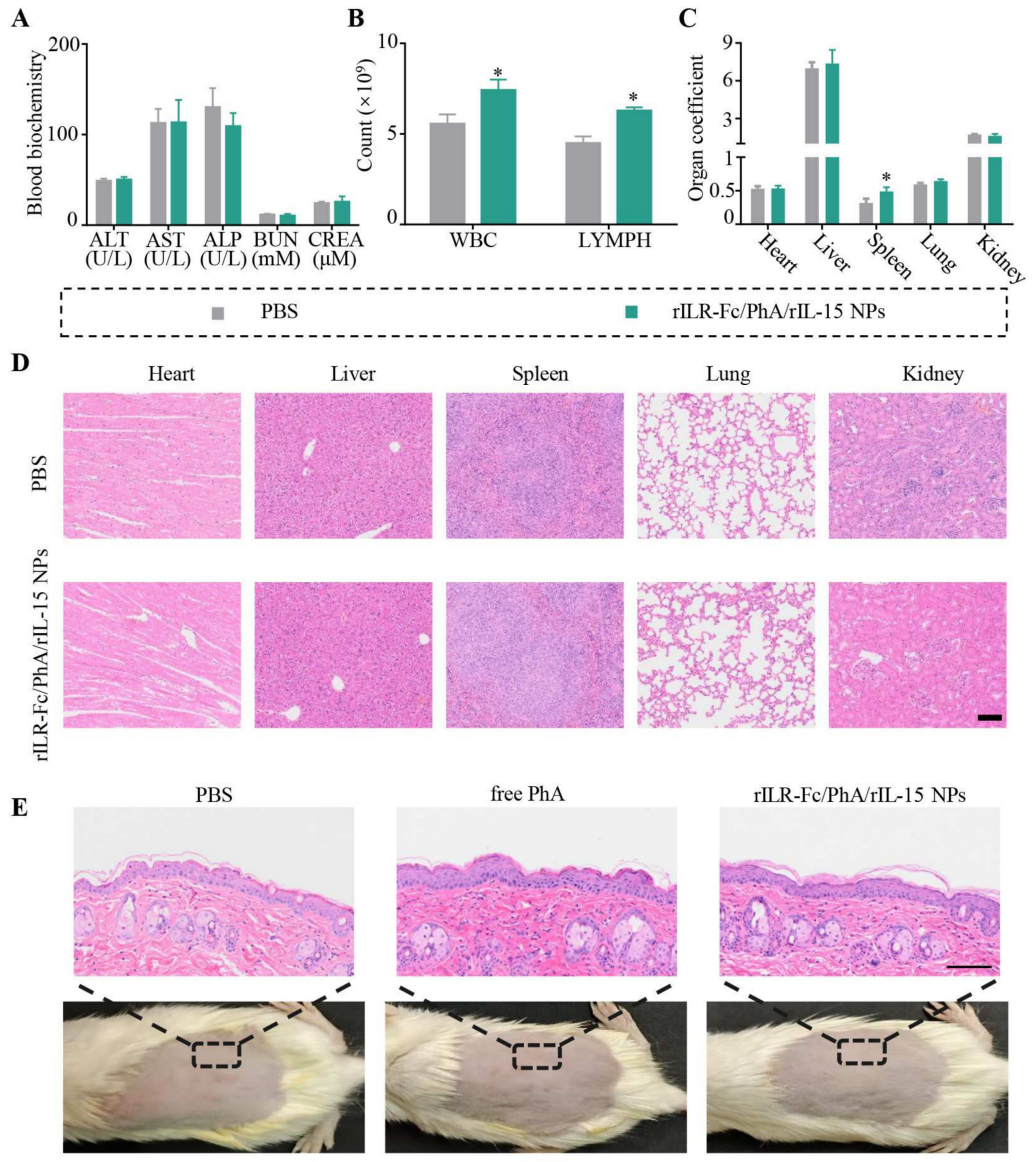
The biosafety evaluation of the rILR-Fc/PhA/rIL-15 NPs. (**A**,**B**) Blood biochemistry (**A**) and hematologic (**B**) analysis after PBS and rILR-Fc/PhA/rIL-15 NP (2.5 mg/kg of PhA and 0.3 mg/kg rIL-15) treatment in ICR mice. Blood biochemistry data including alanine transaminase (ALT), aspartate transaminase (AST), alkaline phosphatase (ALP), blood urea nitrogen (BUN), and creatinine (CREA) were measured. Hematologic analysis included the counts of white blood cells (WBC) and lymphocytes (LYMPH), respectively. Data are means ± SD (*n* = 3). Statistical significance was calculated by unpaired two-tailed *t*-test. * *p* < 0.05 compared with the PBS group. (**C**) Organ coefficient analysis. Data are means ± SD (*n* = 3). Statistical significance was calculated by unpaired two-tailed *t*-test. * *p* < 0.05 compared with the PBS group. (**D**) H&E staining images of the major organs. Scale bar, 100 μm. (**E**) Representative images of the H&E-stained skin following i.v. injection with PBS, free PhA, or rILR-Fc/PhA/rIL-15 NPs. The skin samples were collected at 2 d after xenon light exposure. The dose of PhA was 2.5 mg/kg. Scale bar, 100 μm.

To investigate the biosafety of the rILR-Fc/PhA/rIL-15 NPs in vivo, the liver and renal function was assessed through blood biochemistry tests. No significant changes in alanine transaminase (ALT), aspartate transaminase (AST), blood urea nitrogen (BUN), and creatinine (CREA) were observed in the rILR-Fc/PhA/rIL-15 NPs group compared with the PBS control (Figure 7A and Table S3). Most hematologic parameters did not show significantly changes, apart from the counts of white blood cells (WBC) and lymphocytes (LYMPH) (Figure 7B and Table S3). Additionally, the rILR-Fc/PhA/rIL-15 NPs did not cause significant changes in body weight (Figure S15A) compared with the PBS control. Except for the spleen, all investigated organs’ coefficients were not changed (Figure 7C and Figure S15B). The increase in the organ coefficient of the spleen could be attributed to the promotion of T cell proliferation in the spleen by the rILR-Fc/PhA/rIL-15 NPs. This was in line with a previous report that an IL-15 fusion protein led to lymphocytosis in the blood and splenomegaly [11,12]. Additionally, the rILR-Fc/PhA/rIL-15 NPs did not cause noticeable tissue damage in the major organs analyzed by H&E staining (Figure 7D).

Moreover, the mice in each group were exposed to xenon light (5 mW/cm^2^) for 25 min to evaluate the skin photosensitivity. The results of H&E staining illustrated that the skin of mice treated with rILR-Fc/PhA/rIL-15 NPs or free PhA remained unchanged after light exposure. The separation of the stratum corneum from the skin was observed in all groups, but without necrosis in the epidermis or papillary dermis or other damage (Figure 7E). In addition, the fluorescence intensity in the skin did not show significant differences between the rILR-Fc/PhA/rIL-15 NP group and the free PhA group at 0.5 h or 2 h (Figure S15C,D). All these results demonstrated that the rILR-Fc/PhA/rIL-15 NPs are similar to free PhA, with minimal skin sensitivity [26].

## 4. Conclusions

In this work, we developed co-hitchhiking nanocarriers (rILR-Fc/PhA/rIL-15 NPs) combining PDT and IL-15 immunotherapy. The rILR-Fc loaded PhA based on the high affinity of PhA to IgG-Fc and pre-associated rIL-15 through the receptor–ligand interaction. The rILR-Fc/PhA/rIL-15 NPs significantly prolonged the circulation half-life of rIL-15 but not PhA in the blood. The rILR-Fc/PhA/rIL-15 NPs enhanced the tumor accumulation of PhA and IL-15 through FcRn-mediated endocytosis and micropinocytosis. The co-delivery nanocarrier effectively promoted the maturation of DCs and elicited potent systemic antitumor immunity and long-lasting immune memory against tumor rechallenge, thus exhibiting translational potential for colon cancer therapy.

## Figures and Tables

**Table 1:** Pharmacokinetic parameters of PhA after i.v. administration of free PhA or rILR-Fc/PhA/rIL-15 NPs (2.5 mg/kg of PhA) in mice ^a^.

Parameter	Free PhA	rILR-Fc/PhA/rIL-15 NPs
*t*_1/2_ (h)	1.764 ± 0.1639	1.402 ± 0.1458
AUC_0→n_ (μg/mL·h)	6.489 ± 1.009	9.745 ± 0.4210 *
AUMC (μg/mL·h^2^)	15.97 ± 4.162	14.18 ± 2.267
MRT (h)	2.349 ± 0.3148	1.454 ± 0.1853
*Cl* ((μg)/(μg/mL)/h)	8.129 ± 1.527	5.192 ± 0.2163
*V*_ss_ ((μg)/(μg/mL))	18.20 ± 1.111	7.472 ± 0.7034 **

^a^ Data are presented as means ± SD (*n* = 3). Statistical significance was calculated by unpaired two-tailed *t*-test. * *p* < 0.05 and ** *p* < 0.01 compared with the free PhA group.

**Table 2:** Pharmacokinetic parameters of rIL-15 after i.v. administration of free rIL-15, rILR-Fc/rIL-15, or rILR-Fc/PhA/rIL-15 NPs (0.3 mg/kg of rIL-15) in mice ^a^.

Parameter	rIL-15	rILR-Fc/rIL-15	rILR-Fc/PhA/rIL-15 NPs
*t*_1/2_ (min)	20.24 ± 1.170	58.33 ± 3.180 ***	39.44 ± 4.584 *^,§^
AUC_0→n_ (ng/mL·min) (×10)	664.4 ± 196.1	2227 ± 229.9 **	2732 ± 129.8 ***
AUMC (ng/mL·min^2^) (×10^2^)	544.5 ± 73.17	5644 ± 1084 ****	5102 ± 1417 ****
MRT (min)	9.913 ± 2.956	29.21 ± 6.852	18.80 ± 1.391
*Cl* ((μg)/(ng/mL)/min) (×10^−4^)	5.766 ± 2.519	2.635 ± 0.2533	1.875 ± 0.08779
*V*_ss_ ((μg)/(ng/mL)) (×10^−3^)	6.351 ± 3.348	7.851 ± 2.311	3.550 ± 4.229

^a^ Data are presented as means ± SD (*n* = 3). Statistical significance was calculated by one-way ANOVA with Tukey’s post hoc test. * *p* < 0.05, ** *p* < 0.01, *** *p* < 0.001, and **** *p* < 0.0001 compared with the rIL-15 group; ^§^ *p* < 0.05 compared with the rILR-Fc/rIL-15 group.

## Data Availability

Data will be made available on request.

## Supplementary Materials

The following supporting information can be downloaded at: https://www.mdpi.com/article/10.3390/pharmaceutics17050615/s1, Figure S1: Schematic illustration of the design of pPIC9K-rILR-Fc (A) and mass spectrometric detection of rILR-Fc protein by matrix-assisted laser desorption/ionization time-of-flight mass spectrometry (MALDI-TOF-MS) (B); Figure S2: Preparation and characterization of recombinant proteins; Figure S3: Characterization of rILR-Fc; Figure S4: Characterization of rILR-Fc/PhA/rIL-15 NPs; Figure S5: Biodistribution of PhA from rILR-Fc/PhA/rIL-15 NPs in vivo; Figure S6: Biodistribution of PhA from rILR-Fc/PhA/rIL-15 NPs ex vivo; Figure S7: Cellular uptake and activity of rILR-Fc/PhA/rIL-15 NPs; Figure S8: Dual fluorescence intravital microscopic imaging of rILR-Fc/PhA/rIL-15 NPs; Figure S9: Systemic anticancer immunity of rILR-Fc/PhA/rIL-15 NPs; Figure S10: Representative flow cytometric plots of Figure 5B; Figure S11: Representative flow cytometric plots of Figure 5C,E; Figure S12: Representative flow cytometric plots of Figure 5F and Figure S9; Figure S13: Bioluminescence imaging of mice of Figure 6B; Figure S14: Tumor growth curves of mice of Figure 6C,D; Figure S15: The toxicity evaluation of rILR-Fc/PhA/rIL-15 NPs; Table S1: Antibody used in this study; Table S2: Characterization of rILR-Fc/rIL-15/PhA NPs and rILR-Fc/PhA/ rIL-15 NPs; Table S3: Hematological and biochemical analysis after PBS and rILR-Fc/PhA/rIL-15 NPs (2.5 mg/kg of PhA and 0.3 mg/kg rIL-15) treatment in ICR mice. References [10,13,29] are cited in the Supplementary Materials.
