# Supplementary material for: Interleukin-15Rα-Sushi-Fc Fusion Protein Co-Hitchhikes Interleukin-15 and Pheophorbide A for Cancer Photoimmunotherapy
Source: Pharmaceutics. 2025 May 5;17(5):615. doi: 10.3390/pharmaceutics17050615 (PMC12114846; doi:10.3390/pharmaceutics17050615)
Supplement: Supplementary file 1 [file pharmaceutics-17-00615-s001.zip › pharmaceutics-3551617-supplementary.pdf]

Supplementary Materials

Interleukin-15Rα-sushi-Fc fusion protein co-hitchhikes interleukin-15 and pheophorbide A for cancer photoimmunotherapy

Zhe Li[1,†], Jiaojiao Xu[1,†], Hongzheng Lin[1], Sheng Yu[1], Jingwen Sun[1], Chen Zhang[1], Sihang Zhang[1], Tingting Li[1], Afeng Yang[1], Wei Lu[1,2]*

[1] School of Pharmacy & Minhang Hospital, Key Laboratory of Smart Drug Delivery Ministry of Education & State Key Laboratory of Molecular Engineering of Polymers, Fudan University, 826 Zhangheng Road, Shanghai 201203, China

[2] Quzhou Fudan Institute, 108 Minjiang Avenue, Quzhou 324002, China

* Corresponding author: wlu@fudan.edu.cn

[†] These authors contributed equally to this work.

1.Methods

1.1. Cloning, expression and purification of recombinant proteins

Recombinant interleukin-15 (rIL-15) was produced in *E. coli* BL21 (DE3) cells. The gene encoding N72D mutated interleukin-15 [1] was amplified by the polymerase chain reaction (PCR), and cloned into the pET28a expression vector using Nhe I & Hind III restriction enzymes. Transformed *E. coli* cells were incubated in Luria Bertani medium at 37 °C under kanamycin selection (50 μg/mL). Bacterial growth was monitored by measuring optical density at 600 nm ($OD_{600\ nm}$). When the $OD_{600\ nm}$ reached 0.6, protein expression was induced with 0.5 mM isopropyl-β-D-thiogalactopyranoside (IPTG) for 4 h. Following induction, the cells were harvested by centrifugation and suspended in 50 mM Tris-HCl buffer (pH = 8.0) containing 200 mM NaCl. After sonication, the suspension was centrifuged at 8000 g for 7 min at 4 °C. The pellet was suspended several times in the 50 mM Tris-HCl buffer (pH = 8.0) containing 1% Triton X-100, 2M urea and 200 mM NaCl to remove nonspecifically adsorbed proteins. The pellet was then solubilized in the 50 mM Tris-HCl buffer (pH = 8.0) with 8 M urea and 200 mM NaCl. rIL-15 was refolded by stepwise dialysis [2]. The unfolded protein was gradually refolded by removing urea in steps from 6 to 0 M urea via dialysis. To purify the refolded protein, the filtered protein solution in 20 mM Tris-HCl buffer (pH = 8.0) containing 0.5 M NaCl was applied to a Ni-NTA column (Bestchrom, Shanghai, China) and eluted with a gradient of 0-0.5 M imidazole.

The recombinant interleukin-15Rα-sushi-Fc fusion protein (rILR-Fc) was generated by fusing the sushi domain (ILR, amino acids 1-77) of IL-15Rα [3] to the CH2-CH3 region of human IgG1-Fc. The gene fragment was custom-synthesized by Sangon Biotech (Shanghai, China) and subcloned into a *Pichia pastoris* expression vector. PCR was used to amplify the

gene fragment (ILR-Fc), which was then ligated into the liner pPIC9k vector. The recombinant plasmid pPIC9k-rILR-Fc was linearized by Sac I, transformed into *Pichia pastoris* GS115 cells via electroporation, and filtered by yeast extract peptone dextrose plates containing 4 mg/mL geneticin. The induction steps were performed according to the Pichia expression manual. After 3 days of continuous induction, yeast cells were removed by centrifugation at 8000 g for 10 min. The culture supernatant was then concentrated by a centrifugal filter device (10 kDa), and protein was purified from the cell culture medium by EzScreen Diamond MMC column (Bestchrom, Shanghai, China).

1.2. Confirmation of recombinant proteins and nanoparticles.

Matrix-assisted laser desorption/ionization time-of-flight mass spectrometry (MALDI-TOF-MS) analysis was performed to calculate the molecular mass of rILR-Fc, with sinapic acid selected as the matrix. The released oligosaccharides were analyzed by sodium dodecyl sulfate-polyacrylamide gel electrophoresis (SDS-PAGE). The rILR-Fc was denatured by heating to 100 °C for 10 min. Oligosaccharides were released from denatured rILR-Fc by digestion with PNGase F (Yeasen, Shanghai, China) at 37 °C for 3 h in 50 mM sodium phosphate (pH = 7.5) containing 10% NP-40.

The biological activity of recombinant proteins or nanoparticles were determined by their ability to stimulate the proliferation of M-07e cells. Recombinant wild type IL-15 (rIL-15-wt, 10360-H07E) was obtained from Sino biological (Beijing, China). The commercial whole IL-15Rα fusion protein (rIL-15Rα-Fc, ILA-H5253) was purchased from ACRO Biosystems (Beijing, China). To prepare IL-15 complex protein, rIL-15 or rIL-15-wt was mixed with equimolar amounts of rILR-Fc and rIL-15Rα-Fc, and incubated for 30 min at 37 °C. M-07e

cells (10,000 cells/well) were plated in RPMI-1640 supplemented with 10% fetal bovine serum (FBS) in a 96-well plate and incubated with proteins (rIL-15, rILR-Fc/rIL-15, rIL-15Rα-Fc/rIL-15, rIL-15-wt, rILR-Fc/rIL-15-wt and rIL-15Rα-Fc/rIL-15-wt) at the specified concentration for 72 h at 37 °C. Cell proliferation was determined using the water-soluble tetrazolium 1 (WST-1) cell counting kit (Beyotime, Shanghai, China) following the manufacturer's instructions. Similarity, the biological activity of nanoparticles was determined by incubated with nanoparticles at the specified concentration to stimulate the proliferation of M-07e cells. For the evaluation of biological activity of nanoparticles after photodynamic therapy (PDT) treatment, the nanoparticles were irradiated with a 660 nm diode laser (Cnilaser, Changchun, China) for 10 min at power densities of 50, 80, or 100 mW/cm^2, prior to being used to stimulate the proliferation of M-07e cells.

Affinity was measured by enzyme-linked immunosorbent assay (ELISA). Mouse anti-human IL-15 monoclonal antibody (0.5 μg/mL, MA5-23729, Thermo fisher, Carlsbad, CA, USA) was coated onto ELISA plates to capture rIL-15 (0.2 μg/mL). rILR-Fc or rIL-15Rα-Fc at designated concentrations diluted in 0.1% bovine serum albumin in phosphate buffered saline, was added to plates and incubated at room temperate for 2 h. After washing, horseradish peroxidase (HRP)-conjugated rabbit anti-human IgG-Fc (0.5 μg/mL, 10702-T16-H, Sino biological, Beijing, China) was added, followed by detection using the 3,3',5,5'-tetramethylbenzidine (TMB) substrate kit (Beyotime, Shanghai, China).

1.3 Biosafety analysis in vitro

In human umbilical vein endothelial cells (HUVECs), the biosafety of the formulations was evaluated. Briefly, HUVECs were seeded in a 96-well cell culture plate and incubated at

37 °C in 5% CO_2 for 24 h. Then, the HUVECs were incubated with rILR-Fc/rIL-15 or rILR-Fc/PhA/rIL-15 NPs at different concentration for 2 h. After replaced with fresh medium, the cells were further cultured for additional 24 h followed by the 3-(4,5-dimethylthiazol-2-yl)-2,5-diphenyltetrazolium bromide (MTT) assay. Cell viability was calculated using the following equation.

$$\text{Cell viability (\%)} = \frac{A_{\text{test}} - A_{\text{blank}}}{A_{\text{control}} - A_{\text{blank}}} \times 100\% \quad (1)$$

Supplementary Figures

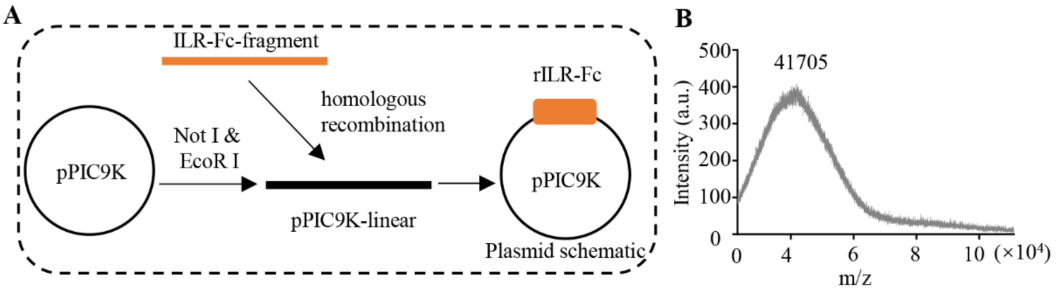

Figure S1. Schematic illustration of the design of pPIC9K-rILR-Fc (**A**) and mass spectrometric detection of rILR-Fc protein by matrix-assisted laser desorption/ionization time-of-flight mass spectrometry (MALDI-TOF-MS) (**B**).

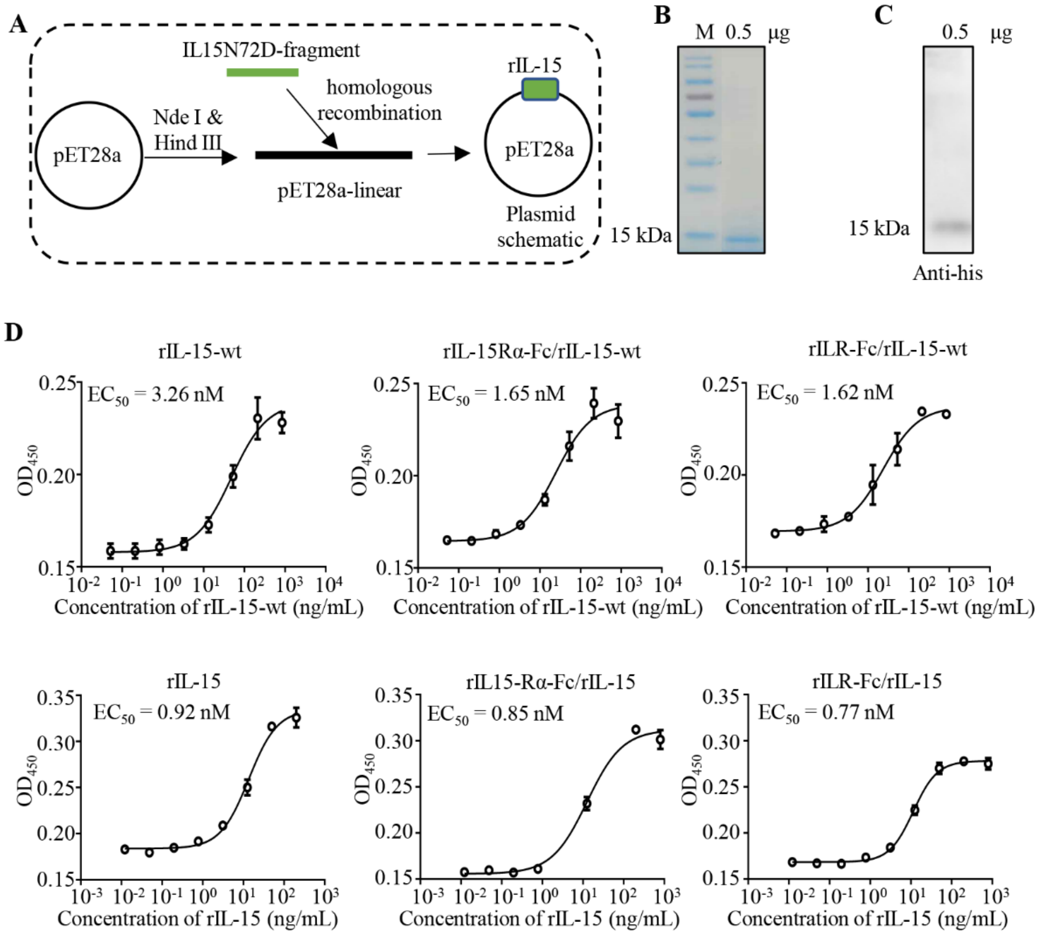

Figure S2. Preparation and characterization of recombinant proteins. (**A**) Schematic illustration of the design of pET28a-rIL-15. rIL-15 purified from E. coli was shown in SDS-PAGE (**B**) and Western blot analysis (**C**). (**D**) The activity of proteins was detected by cell proliferation assay. M-07e cells were incubated with increasing concentrations of proteins for 72 h, followed by WST-1 addition for 4 h. Cell proliferation was quantitated by absorbance at 450 nm. rIL-15-wt, the recombinant wild type IL-15. rIL-15Rα-Fc, the commercial whole IL-15Rα fusion protein. Data are means ± SD (n = 3).

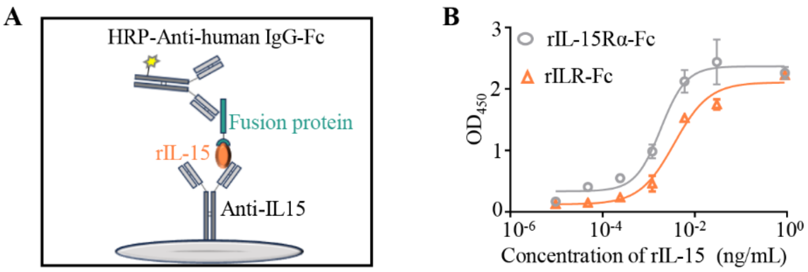

Figure S3. Characterization of rILR-Fc. (**A**) Schematic illustration of the binding affinity analysis by ELISA. (**B**) The binding of increasing concentrations of rIL-15Rα-Fc or rILR-Fc to rIL-15 captured by anti-IL-15 on a 96-well plate was determined by ELISA (n = 3).

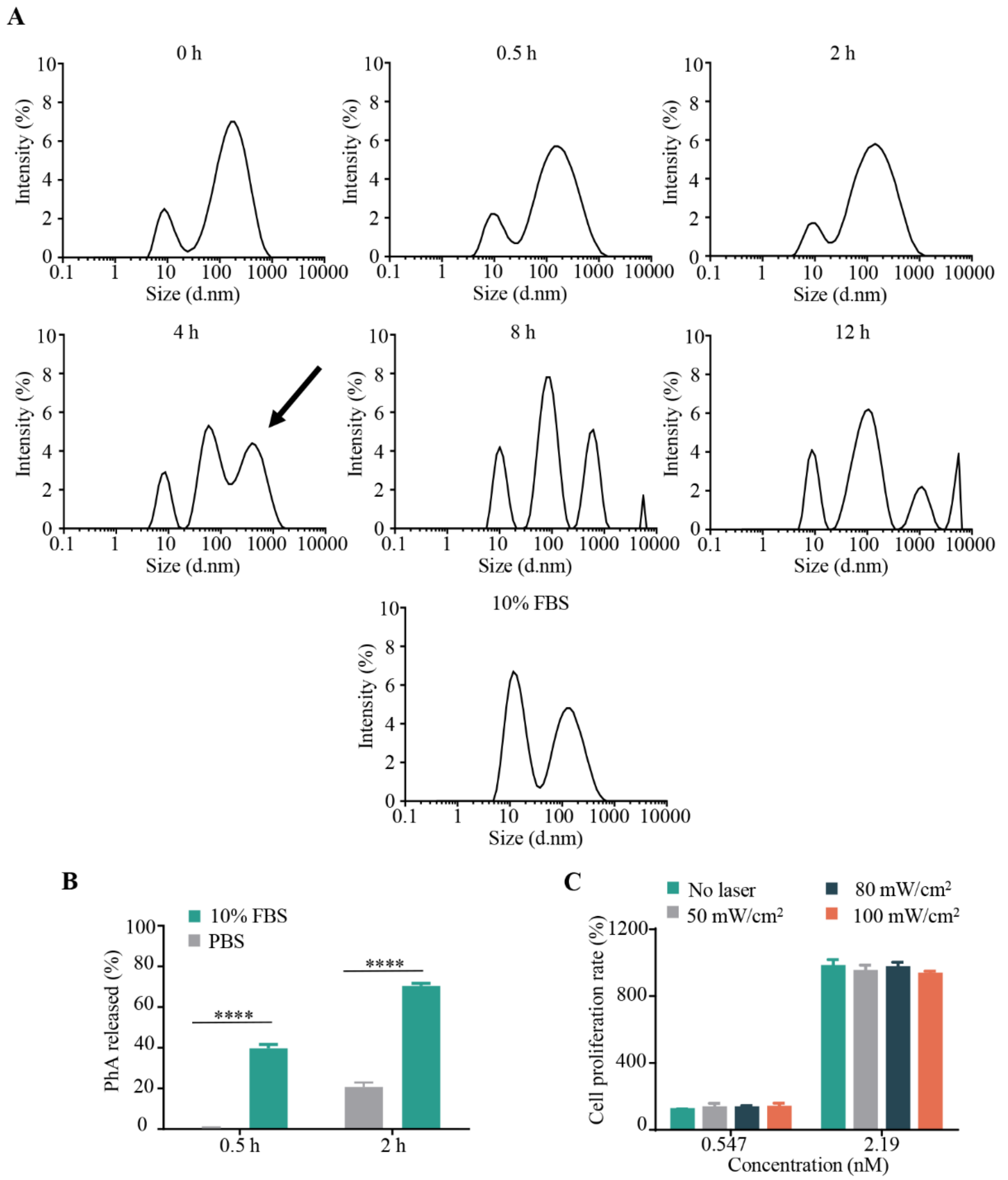

Figure S4. Characterization of rILR-Fc/PhA/rIL-15 NPs. (**A**) Representative size distribution of rILR-Fc/PhA/rIL-15 NPs in PBS with 10% FBS after mixture for different time points. 10% FBS, as control. Black arrow, the aggregation peak of the nanoparticles. (**B**) Cumulative release of PhA from rILR-Fc/PhA/rIL-15 NPs in PBS or 10% FBS at 0.5 h and 2 h. Two-way ANOVA with Holm-Sidak's post hoc test. (n = 3), $^{****}P < 0.0001$. (**C**) The bioactivity of rILR-Fc/PhA/rIL-15 NPs after PDT treatment. rILR-Fc/PhA/rIL-15 NPs containing rIL-15 (0.547 nM and 2.19 nM) were irradiated with laser power densities of 0, 50, 80 and 100 mW/cm^2 for 10 min, respectively. M-07e cells were then incubated with rILR-Fc/PhA/rIL-15 NPs for 72 h, followed by WST-1 addition for 4 h. Cell proliferation was quantitated by absorbance reading at 450 nm. Data are means ± SD (n = 3). One-way ANOVA with Tukey's post hoc test.

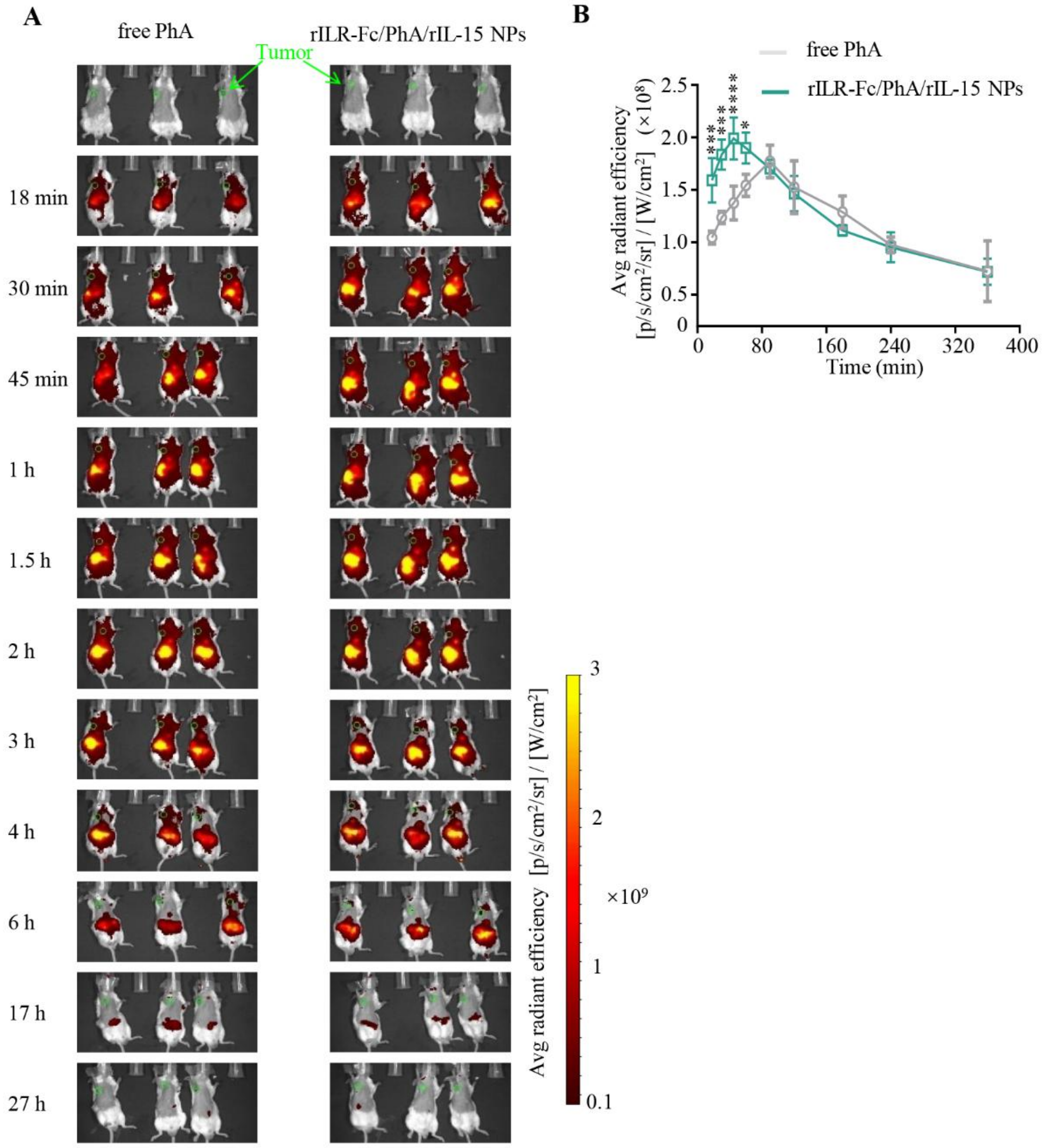

Figure S5. Biodistribution of PhA from rILR-Fc/PhA/rIL-15 NPs in vivo. (**A**) Live fluorescence imaging of PhA distribution in mice bearing s.c. CT26-Luc tumor (green circle) at different time points post the i.v. injection of free PhA or rILR-Fc/PhA/rIL-15 NPs (λ_{ex} = 675 nm, λ_{em} = 720 nm). (**B**) Corresponding fluorescence signal intensity of PhA in the tumor of mice at different time points post-injection of free PhA or rILR-Fc/PhA/rIL-15 NPs. Data are means ± SD (*n* = 3). Statistical significance was calculated by two-way ANOVA with Holm-Sidak's post hoc test. $^{*}P < 0.05$, $^{***}P < 0.001$ and $^{****}P < 0.0001$ compared with the free PhA group.

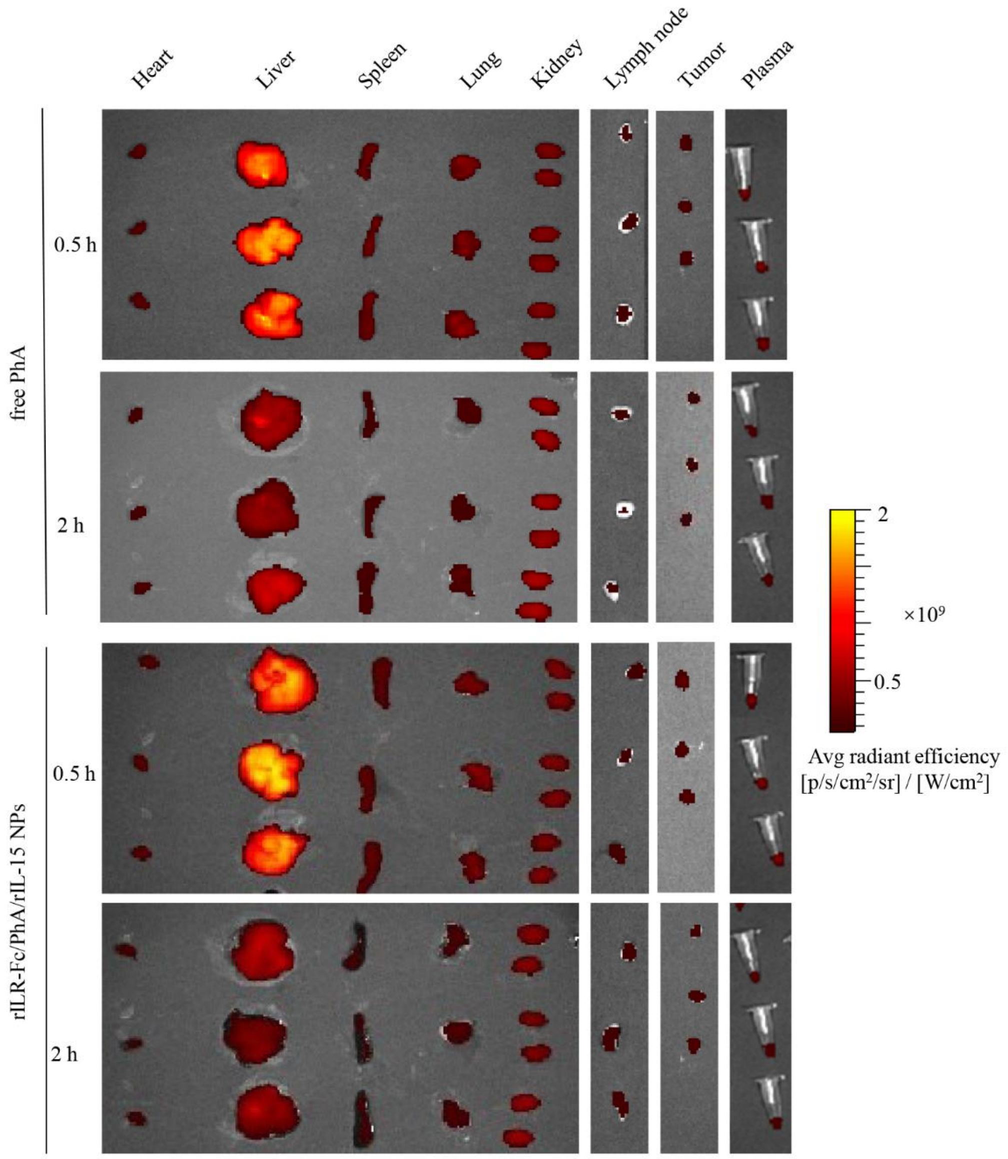

Figure S6. Biodistribution of PhA from rILR-Fc/PhA/rIL-15 NPs ex vivo. The fluorescence imaging of PhA in mice bearing CT26-Luc orthotopic colon tumor model at 0.5 h or 2 h after the i.v. injection of free PhA or rILR-Fc/PhA/rIL-15 NPs (λ_{ex} = 675 nm, λ_{em} = 720 nm). The volume of each plasma sample was 20 μL.

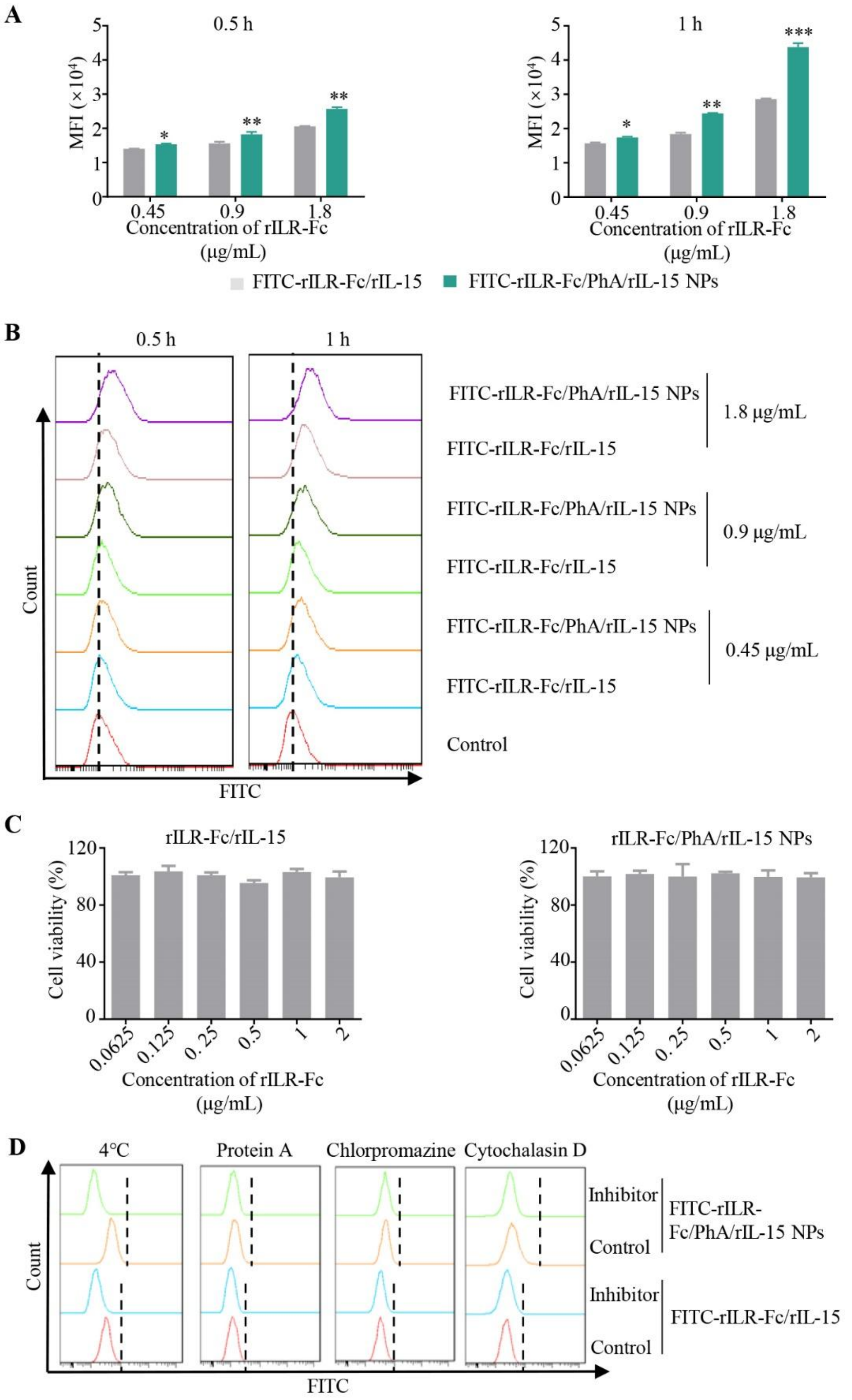

Figure S7. Cellular uptake and activity of rILR-Fc/PhA/rIL-15 NPs. (**A**) Cellular uptake of FITC-labeled rILR-Fc/rIL-15 or rILR-Fc/PhA/rIL-15 NPs by human umbilical vein endothelial cells (HUVECs) was analyzed by flow cytometry at different concentrations or time points. MFI, mean fluorescence intensity. Two-way ANOVA with Holm-Sidak's post hoc test. Data are means ± SD (n = 3). $^{*}P < 0.05$, $^{**}P < 0.001$ and $^{***}P < 0.001$ compared with the FITC-rILR-

Fc/rIL-15 group. (**B**) Representative flow cytometry histograms of cellular uptake. (**C**) Cell viability of HUVECs incubated with rILR-Fc/rIL-15 or rILR-Fc/PhA/rIL-15 NPs was analyzed by MTT assay (n = 3). (**D**) Representative flow cytometry histograms of cellular uptake in the presence of various endocytosis inhibitors.

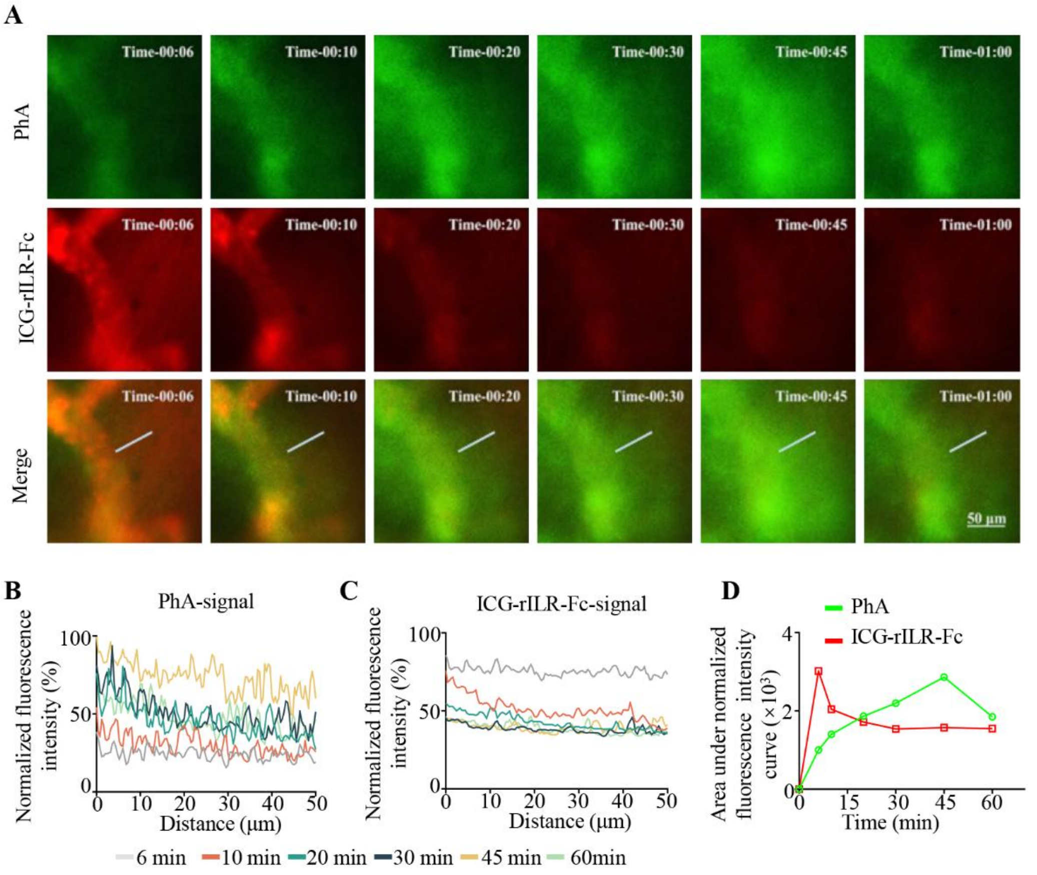

Figure S8. Dual fluorescence intravital microscopic imaging of rILR-Fc/PhA/rIL-15 NPs. (**A**) Dual fluorescence intravital microscopic imaging of PhA (green) and ICG-rILR-Fc (red) at different time points following injection into the orthotopic CT26-Luc tumor. Bars, 50 μm. (**B**,**C**) Fluorescence intensity curves of PhA (**B**) and ICG-rILR-Fc (**C**) at various time points, measured as a function of the distance from the blood vessel in a representative region marked by the white line in (**A**). (**D**) The area under the normalized fluorescence intensity-distance curve (AUNFIC) of ICG-rILR-Fc or PhA at different times post-injection.

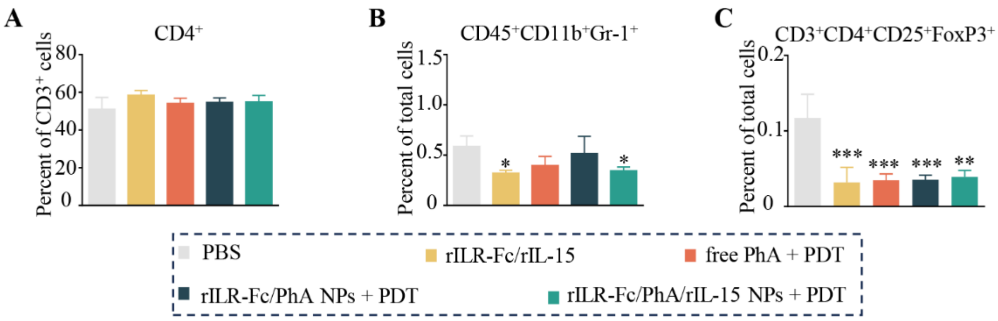

Figure S9. Systemic anticancer immunity of rILR-Fc/PhA/rIL-15 NPs. (**A,B**) Quantitative analysis of $CD4^+$ T cells (A) and $CD45^+CD11b^+Gr\text{-}1^+$ cells (B) in the spleen. Representative flow cytometry plots are shown in Figure S12A. (**C**) Quantitative analysis of $CD3^+CD4^+CD25^+FoxP3^+$ cells in tumor. Representative flow cytometry plots are shown in Figure S12A. One-way ANOVA with Dunnett's post hoc test. Data are means ± SD (n = 3). $^{*}P < 0.05$, $^{**}P < 0.01$ and $^{***}P < 0.001$ compared with the PBS group.

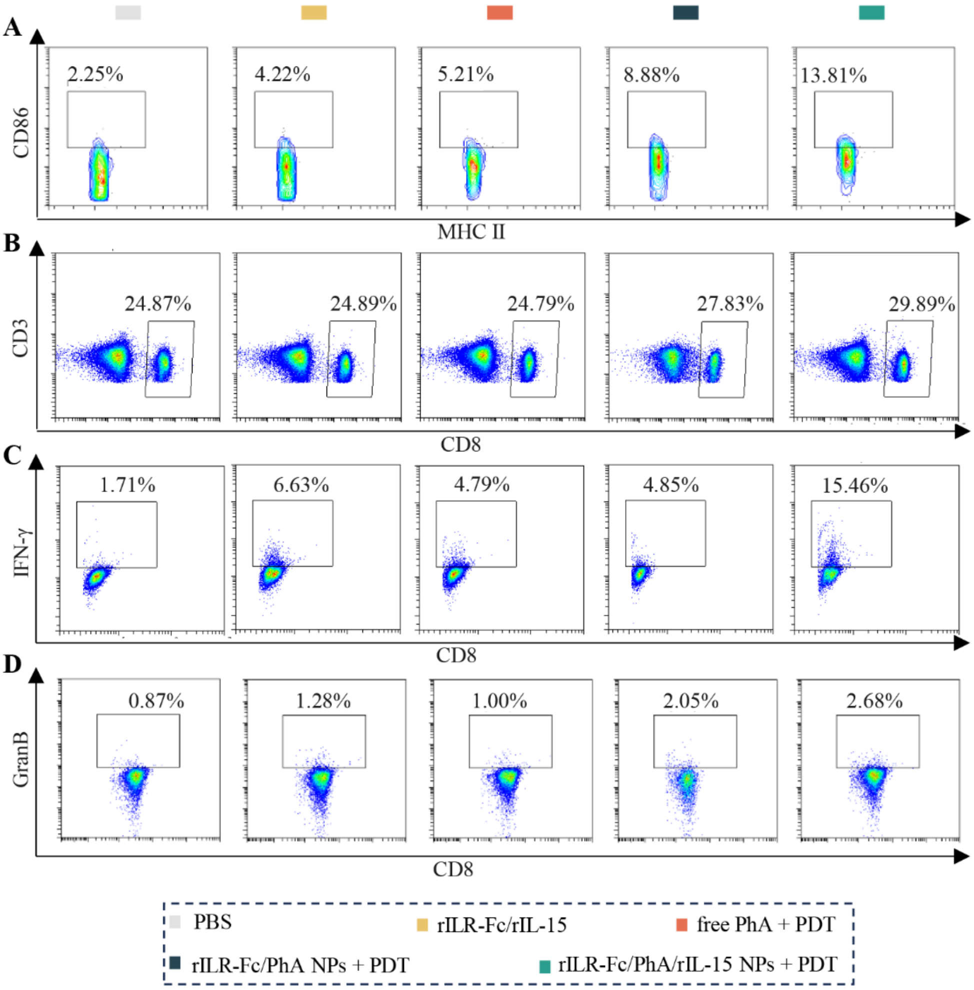

Figure S10. Representative flow cytometric plots of Figure 5B. The representative plots of $CD45^{+}CD11c^{+}MHCII^{hi}CD86^{hi}$ cells (**A**), $CD8^{+}$ T cells (**B**), $CD8^{+}IFN\text{-}\gamma^{+}$ T cells (**C**) and $CD8^{+}GranB^{+}$ T cells (**D**) in DLN.

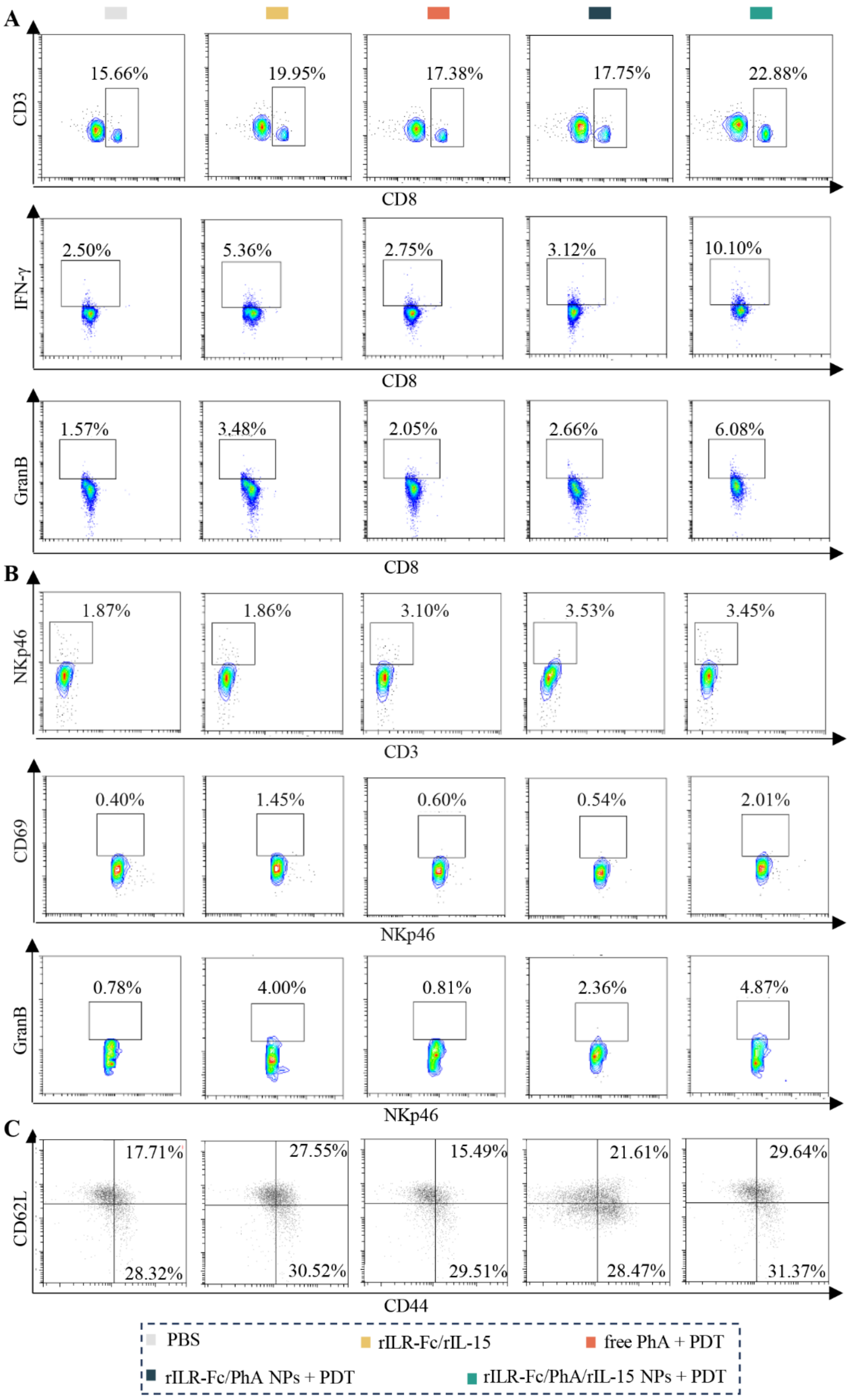

Figure S11. Representative flow cytometric plots of Figure 5C,E. (**A**) The representative plots of CD8^{+} T cells, CD8^{+}IFN-γ^{+} T cells and CD8^{+}GranB^{+} T cells in spleen. (**B**) The representative plots of CD45^{+}CD3^{-}NKp46^{+} cells, CD45^{+}CD3^{-}NKp46^{+}CD69^{+} cells and CD45^{+}CD3^{-}NKp46^{+}GranB^{+} cells in spleen. (C) The representative plots of CD8^{+}CD44^{+} T cells, CD8^{+}CD44^{+}CD62L^{-} T cells and CD8^{+}CD44^{+}CD62L^{+} T cells in the spleen.

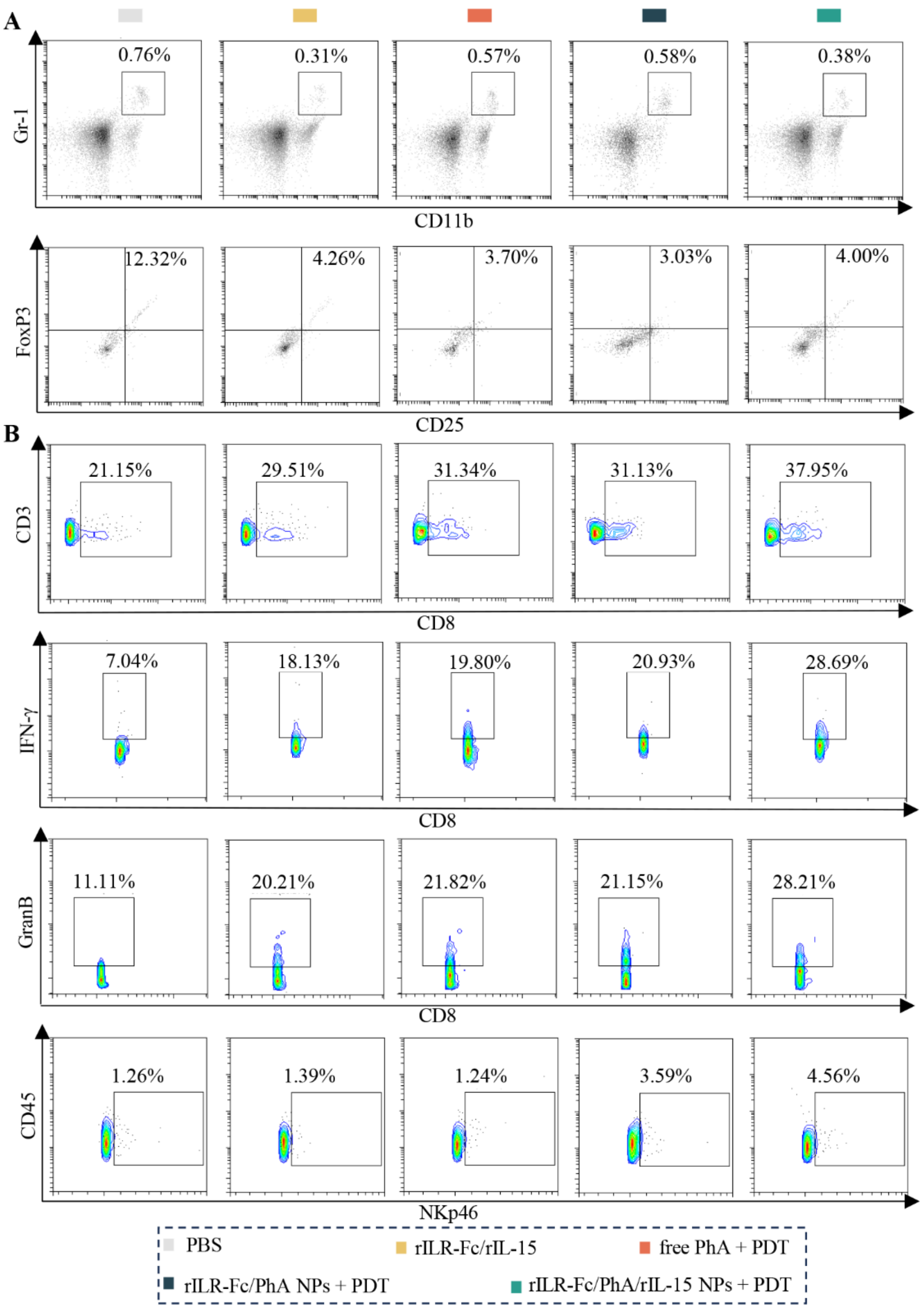

Figure S12. Representative flow cytometric plots of Figures 5F and S9. (**A**) The representative plots of $CD45^+CD11b^+Gr\text{-}1^+$ cells in spleen and $CD3^+CD4^+CD25^+FoxP3^+$ cells in tumor. (**B**) The representative plots of $CD8^+$ T cells, $CD8^+IFN\text{-}\gamma^+$ T cells, $CD8^+GranB^+$ T cells and $CD45^+CD3^-NKp46^+$ cells in tumor.

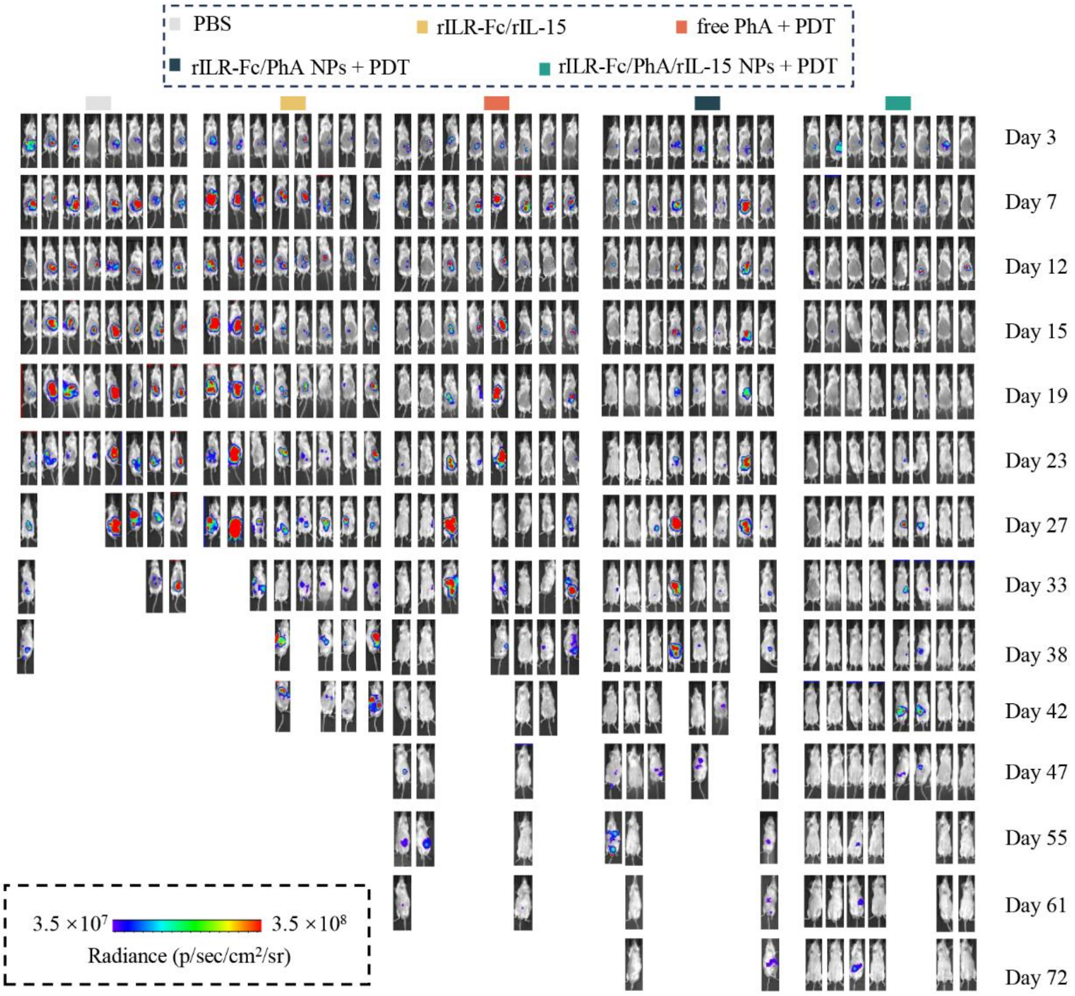

Figure S13. Bioluminescence imaging of mice of Figure 6B.

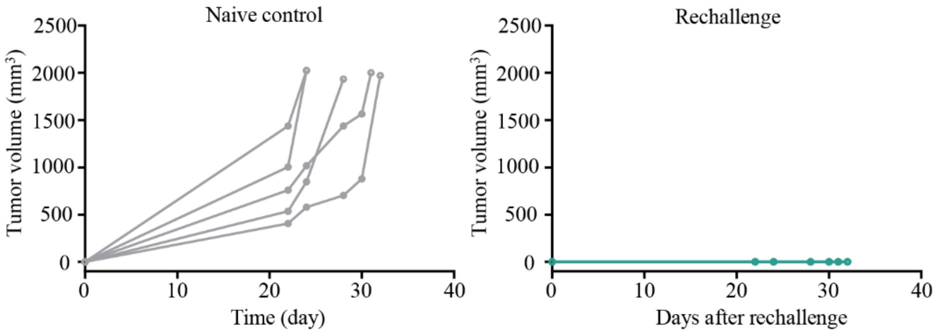

Figure S14. Tumor growth curves of mice of Figure 6C,D.

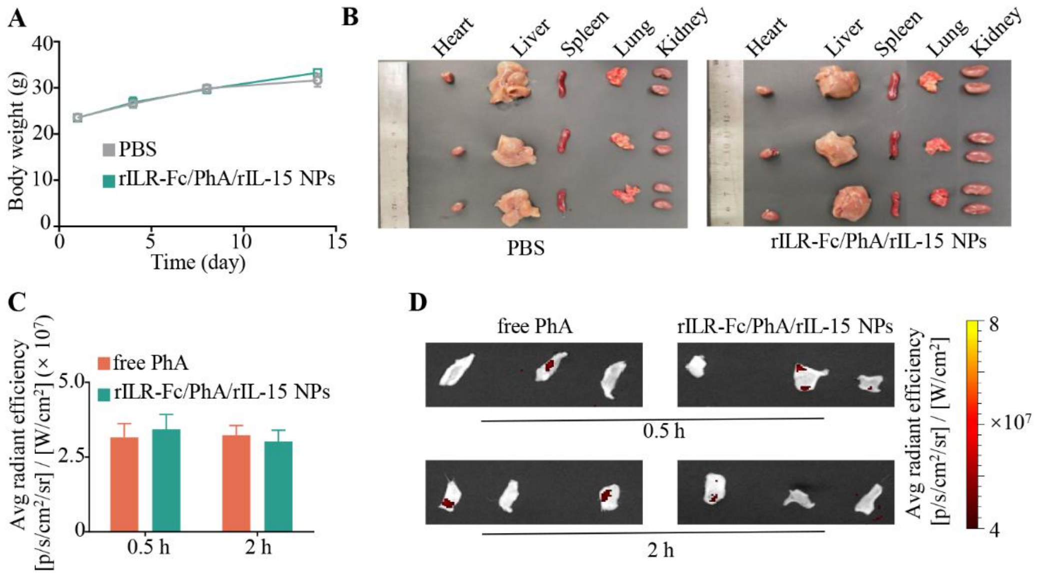

Figure S15. The toxicity evaluation of rILR-Fc/PhA/rIL-15 NPs. (**A**) Body weight changes in ICR mice after PBS and rILR-Fc/PhA/rIL-15 NPs (2.5 mg/kg of PhA and 0.3 mg/kg rIL-15) treatment (n = 3). (**B**) Photographs of organs of Fig. 7C. (**C,D**) Quantification (**C**) and fluorescence imaging (**D**) of PhA in the skin of BALB/c mice at 0.5 h or 2 h following i.v. injection of free PhA and rILR-Fc/PhA/rIL-15 NPs (2.5 mg/kg of PhA). Data are means ± SD (n = 3).

Table S1. Antibody used in this study.

Antibody	Catalog number	Company
Anti-mouse CD3-APC	170031-81	Invitrogen
Anti-mouse IFN-γ- PerCP-Cyanine5.5	45-7311-80	Invitrogen
Anti-mouse CD45-APC	17-0451-82	Invitrogen
Anti-mouse CD3-PE	12-0031-82	Invitrogen
Anti-mouse CD25-PE	12-0251-82	Invitrogen
Anti-mouse Gr-1-PE	12-5931-81	Invitrogen
Anti-mouse CD45-PE	12-0451-83	Invitrogen
Anti-mouse CD11c-PerCP-Cyanine5.5	45-0114-82	Invitrogen
Anti-mouse MHC II-APC	17-5321-82	Invitrogen
Anti-mouse CD86-FITC	11-0862-82	Invitrogen
Anti-mouse FoxP3-PerCP-Cyanine5.5	45-5773-82	Invitrogen
Anti-mouse CD8-FITC	11-0081-82	Invitrogen
Anti-mouse CD44-PerCP-Cyanine5.5	45-0441-82	Invitrogen
Anti-mouse CD69-FITC	11-0691-82	Invitrogen
Anti-mouse CD4-APC	17-0041-82	Invitrogen
Anti-mouse IFN-γ-APC	17-7311-82	Invitrogen
Rat IgG2b kappa isotype control-PerCP-Cyanine5.5	45-4031-80	Invitrogen
Rat IgG2b kappa isotype control-PE	12-4031-82	Invitrogen
Rat IgG1 kappa isotype control-PE	12-4301-82	Invitrogen
Rat IgG1 kappa isotype control-APC	17-4301-82	Invitrogen
Armenian Hamster IgG isotype control-PerCP-Cyanine5.5	45-4888-80	Invitrogen
Rat IgG2a kappa isotype control-FITC	11-4321-80	Invitrogen
Rat IgG2a kappa isotype control-APC	17-4321-81	Invitrogen
Mouse IgG1 kappa isotype control-PE	12-4714-82	Invitrogen
Armenian Hamster IgG isotype control-APC	17-4888-82	Invitrogen

Anti-mouse CD8-PE	100708	Biolegend
Anti-mouse granzyme B-FITC	396404	Biolegend
Anti-rat IgG1 kappa isotype control-FITC	400405	Biolegend
Anti-mouse NKp46-PerCP-Cyanine5.5	137609	Biolegend
Anti-rat IgG2a kappa isotype control-PerCP-Cyanine5.5	400531	Biolegend
Anti-mouse CD45-FITC	103107	Biolegend
Anti-mouse CD11b-APC	101212	Biolegend
Anti-rat IgG2b kappa isotype control-APC	400611	Biolegend
Anti-mouse CD4-FITC	100405	Biolegend
Anti-mouse CD4-PE/Cyanine7	100527	Biolegend
Rat IgG2a kappa isotype control-PE/Cyanine7	400521	Biolegend

Table S2. Characterization of rILR-Fc/rIL-15/PhA NPs and rILR-Fc/PhA/ rIL-15 NPs [a].

Types	rILR-Fc/PhA/rIL-15 NPs	rILR-Fc/rIL-15/PhA NPs
Size (nm)	24.48 ± 0.47	23.07 ± 0.58
EE% (PhA)	98.94 ± 0.95	97.30 ± 1.06
EE% (rIL-15)	99.50 ± 0.02	99.20 ± 0.04

[a] Data expressed as means ± SD (*n* = 3).

Table S3. Hematological and biochemical analysis after PBS and rILR-Fc/PhA/rIL-15 NPs (2.5 mg/kg of PhA and 0.3 mg/kg rIL-15) treatment in ICR mice [a].

Item	Reference range	PBS	ILR-Fc/PhA/rIL-15 NPs
WBC (10^9/L)	4.4-14.01	5.57 ± 0.51	7.43 ± 0.57*
LYMPH (10^9/L)	2.06-10.01	4.50 ± 0.36	6.30 ± 0.17*
MONO (10^9/L)	0.18-0.32	0.17 ± 0.06	0.17 ± 0.06
NEUT (10^9/L)	0.53-5.17	0.90 ± 0.26	0.97 ± 0.35
LYMPH (%)	37.50-85.01	80.73 ± 4.24	84.73 ± 4.40
MONO (%)	2.84-13.09	3.17 ± 0.32	2.47 ± 0.72
NEUT (%)	8.74-55.68	16.10 ± 3.96	12.80 ± 3.84
RBC (10^{12}/L)	7.31-12.27	8.68 ± 0.40	8.97 ± 0.85
HGB (g/L)	119-184	146.00 ± 6.56	142 .33 ± 1.53
HCT (%)	39.7-74.7	45.20 ± 2.20	46.73 ± 4.69
MCV (fL)	46.5-69	52.1 ± 0.17	52.17 ± 0.49
MCH (pg)	13.1-18.8	16.77 ± 0.06	15.93 ± 1.59
MCHC (g/L)	302-353	322.67 ± 2.52	306.33 ± 31.18
RDW (%)	15.1-18.9	13.67 ± 1.36	14.57 ± 0.58
PLT (10^9/L)	736-2374	973.67 ± 167.70	1848.67 ± 699.97
MPV (fL)	4.3-5.8	5.87 ± 0.06	6.07 ± 0.72
ALT (U/L)	28-64	48.56 ± 2.92	49.97 ± 3.32
AST (U/L)	47-120	112.41 ± 16.03	113.14 ± 25.12
ALP (U/L)	22.52-474.35	12.99 ± 2.13	10.89 ± 1.49
BUN (mg/dl)	16-22	31.25 ± 3.57	27.95 ± 6.24
CREA (μmol/L)	10.91-85.09	23.84 ± 2.05	25.50 ± 6.25

[a] Blood levels of various hematological parameters, including white blood cells (WBC), lymphocytes (LYMPH), monocytes (MONO), neutrophils (NEUT), red blood cells (RBC), hemoglobin (HGB), hematocrit (HCT), mean corpuscular volume (MCV), mean corpuscular hemoglobin (MCH), mean corpuscular hemoglobin concentration (MCHC), red cell volume distribution width (RDW), platelets (PLT) and mean platelet volume (MPV) were analyzed.

Serum biochemistry data, including blood urea nitrogen (BUN), creatinine (CREA), aspartate transaminase (AST), alanine transaminase (ALT) and alkaline phosphatase (ALP) were also measured. The reference ranges for these hematological and biochemical parameters in healthy ICR mice were obtained from Charles River Laboratories (http://www.criver.com/). Statistical significance was calculated by unpaired two-tailed *t*-test, $^{*}P < 0.05$ compared with the PBS group.

References

1. Zhu, X., W.D. Marcus, W. Xu, H.I. Lee, et al., Novel human interleukin-15 agonists, J. Immunol, 2009, 183, 3598-3607.

2. Umetsu, M., K. Tsumoto, M. Hara, K. Ashish, et al., How additives influence the refolding of immunoglobulin-folded proteins in a stepwise dialysis system, J. Biol. Chem., 2003, 278, 8979-8987.

3. Desbois, M., C. Béal, M. Charrier, B. Besse, et al., IL-15 superagonist RLI has potent immunostimulatory properties on NK cells: implications for antimetastatic treatment, J. Immunother. Cancer., 2020, 8, e000632.
